# The Transcription Factor Encyclopedia

**DOI:** 10.1186/gb-2012-13-3-r24

**Published:** 2012-03-29

**Authors:** Dimas Yusuf, Stefanie L Butland, Magdalena I Swanson, Eugene Bolotin, Amy Ticoll, Warren A Cheung, Xiao Yu Cindy Zhang, Christopher TD Dickman, Debra L Fulton, Jonathan S Lim, Jake M Schnabl, Oscar HP Ramos, Mireille Vasseur-Cognet, Charles N de Leeuw, Elizabeth M Simpson, Gerhart U Ryffel, Eric W-F Lam, Ralf Kist, Miranda SC Wilson, Raquel Marco-Ferreres, Jan J Brosens, Leonardo L Beccari, Paola Bovolenta, Bérénice A Benayoun, Lara J Monteiro, Helma DC Schwenen, Lars Grontved, Elizabeth Wederell, Susanne Mandrup, Reiner A Veitia, Harini Chakravarthy, Pamela A Hoodless, M Michela Mancarelli, Bruce E Torbett, Alison H Banham, Sekhar P Reddy, Rebecca L Cullum, Michaela Liedtke, Mario P Tschan, Michelle Vaz, Angie Rizzino, Mariastella Zannini, Seth Frietze, Peggy J Farnham, Astrid Eijkelenboom, Philip J Brown, David Laperrière, Dominique Leprince, Tiziana de Cristofaro, Kelly L Prince, Marrit Putker, Luis del Peso, Gieri Camenisch, Roland H Wenger, Michal Mikula, Marieke Rozendaal, Sylvie Mader, Jerzy Ostrowski, Simon J Rhodes, Capucine Van Rechem, Gaylor Boulay, Sam WZ Olechnowicz, Mary B Breslin, Michael S Lan, Kyster K Nanan, Michael Wegner, Juan Hou, Rachel D Mullen, Stephanie C Colvin, Peter John Noy, Carol F Webb, Matthew E Witek, Scott Ferrell, Juliet M Daniel, Jason Park, Scott A Waldman, Daniel J Peet, Michael Taggart, Padma-Sheela Jayaraman, Julien J Karrich, Bianca Blom, Farhad Vesuna, Henriette O'Geen, Yunfu Sun, Richard M Gronostajski, Mark W Woodcroft, Margaret R Hough, Edwin Chen, G Nicholas Europe-Finner, Magdalena Karolczak-Bayatti, Jarrod Bailey, Oliver Hankinson, Venu Raman, David P LeBrun, Shyam Biswal, Christopher J Harvey, Jason P DeBruyne, John B Hogenesch, Robert F Hevner, Christophe Héligon, Xin M Luo, Marissa Cathleen Blank, Kathleen Joyce Millen, David S Sharlin, Douglas Forrest, Karin Dahlman-Wright, Chunyan Zhao, Yuriko Mishima, Satrajit Sinha, Rumela Chakrabarti, Elodie Portales-Casamar, Frances M Sladek, Philip H Bradley, Wyeth W Wasserman

**Affiliations:** 1 Department of Medical Genetics, Faculty of Medicine, Centre for Molecular Medicine and Therapeutics, Child and Family Research Institute, University of British Columbia, 950 West 28th Avenue, Vancouver, British Columbia V5Z 4H4, Canada; 2 Evaluation and Research Services, Fraser Health Authority, 300 - 10334 152A Street, Surrey, British Columbia V3R 7P8, Canada; 3 Children's Hospital Oakland Research Institute, 5700 Martin Luther King Junior Way, Oakland, CA 94609-1809, USA; 4 Computational Biology Program, Public Health Sciences Division, Fred Hutchinson Cancer Research Center, 1100 Fairview Avenue North, Seattle, WA 98109, USA; 5 Department of Bioinformatics, Centre for Molecular Medicine and Therapeutics, Child and Family Research Institute, University of British Columbia, 950 West 28th Avenue, Vancouver, British Columbia V5Z 4H4, Canada; 6 Department of Biology, University of Western Ontario, 1151 Richmond Street, London, Ontario N6A5B7, Canada; 7 Genetics Program, Centre for Molecular Medicine and Therapeutics, Child and Family Research Institute, University of British Columbia, 950 West 28th Avenue, Vancouver, British Columbia V5Z 4H4, Canada; 8 Cell Biology and Neuroscience, Institute of Integrated Genome Biology, University of California at Riverside, 2115 Biological Sciences Building, Riverside, CA 92521, USA; 9 SIMOPRO, Laboratory of Life Sciences (Laboratoire de Sciences du Vivant), CEA (Commissariat à l'Énergie Atomique), Gif-sur-Yvette, Saclay, Île-de-France 91191, France; 10 Department Endocrinology, Metabolism and Cancer, INSERM (Unité 1016), Institut Cochin, 24 Rue du Faubourg Saint Jacques, Paris, Île-de-France 75014, France; 11 Institut für Zellbiologie, Universitätsklinikum Essen, Universität Duisburg-Essen, Hufelandstrasse 55, Essen, Nordrhein-Westfalen 45122, Germany; 12 Department of Surgery and Cancer, Division of Cancer, Imperial College London, Du Cane Road, London, London W12 0NN, UK; 13 Centre for Oral Health Research, School of Dental Sciences, Newcastle University, Medical School, Framlington Place, Newcastle upon Tyne, Tyne and Wear NE2 4BW, UK; 14 Department of Development and Differentiation, Centro de Biologia Molecular Severo Ochoa (CBMSO), Consejo Superior de Investigaciones Científicas (CSIC) and CIBER de Enfermedades Raras (CIBERER), Nicolas Cabrera 1, Cantoblanco, Madrid, Madrid 28049, Spain; 15 Division of Reproductive Health, Warwick Medical School, University of Warwick, Clifford Bridge Road, Coventry, West Midlands CV2 2DX, UK; 16 Neurobiologia Molecular Celular y del desarrollo, Centro de Biologia Molecular Severo Ochoa (CBMSO), Centro de Biologia Molecular Severo Ochoa and CIBER de Enfermedades Raras (CIBERER), Nicolas Cabrera 1, Cantoblanco, Madrid, Madrid 28049, Spain; 17 Department of Molecular and Cellular Pathology, Institut Jacques Monod, Université Paris Diderot (Paris 7), 15 rue Hélène Brion, Paris, Île-de-France 75013, France; 18 Department of Biochemistry and Molecular Biology, University of Southern Denmark, Campusvej 55, Odense, Region Syddanmark 5230, Denmark; 19 Terry Fox Laboratory, BC Cancer Agency, Provincial Health Services Authority, 675 West 10th Avenue, Vancouver, British Columbia V5Z 1L3, Canada; 20 Molecular and Cellular Pathology Program, Institut Jacques Monod, Université Paris Diderot (Paris 7), 15 rue Hélène Brion, Paris, Île-de-France 75013, France; 21 Eppley Institute for Research in Cancer and Allied Diseases, University of Nebraska Medical Center, University of Nebraska, 985950 Nebraska Medical Center, Omaha, NE 68198-5950, USA; 22 Department of Molecular Experimental Medicine, Scripps Research Institute, 10550 North Torrey Pines Road, La Jolla, CA 92037, USA; 23 Departments of Molecular and Experimental Medicine and Immunology and Microbial Sciences (MEM 131), Scripps Research Institute, 10550 North Torrey Pines Road, La Jolla, CA 92037, USA; 24 Nuffield Department of Clinical Laboratory Sciences, John Radcliffe Hospital, Oxford NIHR Biomedical Research Centre, University of Oxford, Level 4 Academic Block, John Radcliffe Hospital, Headington, Oxford, Oxfordshire OX3 9DU, UK; 25 Department of Pediatrics, College of Medicine, University of Illinois at Chicago, 840 South Wood Street (M/C 856), Chicago, IL 60612, USA; 26 Department of Medicine/Hematology, Stanford University School of Medicine, Stanford University, 875 Blake Wilbur Drive, Stanford, CA 94305, USA; 27 Department of Medicine, University of Bern, Hochschulstrasse 4, Bern, Bern-Mittelland CH-3012, Switzerland; 28 Department of Oncology, Sidney Kimmel Comprehensive Cancer Center at Johns Hopkins, Johns Hopkins University School of Medicine, 1650 Orleans Street Room 530, Baltimore, MD 21237, USA; 29 Eppley Institute for Research in Cancer and Allied Diseases, University of Nebraska Medical Center, University of Nebraska, 986805 Nebraska Medical Center, Omaha, NE 68198-6805, USA; 30 Institute of Experimental Endocrinology and Oncology (IEOS), CNR - National Research Council, via Pansini 5, Naples, Naples 80131, Italy; 31 Department of Biochemistry and Molecular Biology, Norris Comprehensive Cancer Center, University of Southern California, 1450 Biggy Street, Los Angeles, CA 90089, USA; 32 Department of Molecular Cancer Research, University Medical Center Utrecht, Utrecht University, Universiteitsweg 100, Utrecht, Utrecht 3584 CG, The Netherlands; 33 Nuffield Department of Clinical Laboratory Sciences, Medical Sciences Division, University of Oxford, Level 4 Academic Block, John Radcliffe Hospital, Headington, Oxford, Oxfordshire OX3 9DU, UK; 34 Molecular Targeting in Breast Cancer research unit, Institute for Research in Immunology and Cancer, Université de Montréal, 2950 Chemin de Polytechnique, Montréal, Québec H3T 1J4, Canada; 35 Institut de Biologie de Lille, Institut Pasteur de Lille, Centre National de la Recherche Scientifique (CNRS) UMR 8161, 1 Rue du Pr Calmette, Lille, Nord-Pas-de-Calais 59021, France; 36 Department of Cellular and Integrative Physiology, Indiana University School of Medicine, Indiana University-Purdue University Indianapolis, 635 Barnhill Drive, Indianapolis, IN 46202, USA; 37 Department of Physiological Chemistry, University Medical Centre Utrecht, Utrecht University, Universiteitsweg 100, Utrecht, Utrecht 3584 CG, The Netherlands; 38 Department of Biochemistry, School of Medicine, Universidad Autonoma de Madrid, Arzobispo Morcillo, 4, Madrid, Madrid 28029, Spain; 39 Institute of Physiology, Zurich Center for Integrative Human Physiology, University of Zurich, Winterthurerstrasse 190, Zurich, Zurich CH-8057, Switzerland; 40 Department of Oncological Genetics, Medical Center of Postgraduate Education, Maria Sklodowska-Curie Memorial Cancer Center and Institute of Oncology, Roentgena 5, Warsaw, Mazovia 02-781, Poland; 41 Department of Biochemistry, Institute for Research in Immunology and Cancer, Université de Montréal, PO Box 6128, Station Centre-Ville, Montréal, Québec H3C 3J7, Canada; 42 Department of Biochemistry, Institute for Research in Immunology and Cancer, Université de Montréal, 2950 Chemin de Polytechnique, Montréal, Québec H3T 1J4, Canada; 43 Department of Biology, School of Science, Indiana University-Purdue University Indianapolis, LD222, 402 North Blackford Street, Indianapolis, IN 46202, USA; 44 Department of Medicine, Cancer Center, Massachusetts General Hospital, Harvard Medical School, 13th Street, Building 149, Room 7.103, Charlestown, MA 02129, USA; 45 Department of Biochemistry, School of Molecular and Biomedical Science, University of Adelaide, North Terrace, Adelaide, South Australia 5005, Australia; 46 Department of Pediatrics and Biochemistry and Molecular Biology, Research Institute for Children, Children's Hospital at New Orleans, Louisiana State University Health Sciences Center, 200 Henry Clay Avenue, New Orleans, LA 70118, USA; 47 Departments of Pediatrics and Genetics, Research Institute for Children, Children's Hospital at New Orleans, Louisiana State University Health Sciences Center, 200 Henry Clay Avenue, New Orleans, LA 70118, USA; 48 Department of Pathology and Molecular Medicine, Queen's Cancer Research Institute, Queen's University, 18 Stuart Street, Botterell Hall, Kingston, Ontario K7L 3N6, Canada; 49 School of Medicine, Institut fuer Biochemie, Emil-Fischer-Zentrum, Friedrich-Alexander Universitaet Erlangen-Nuernberg, Fahrstrasse 17, Erlangen, Bavaria 91096, Germany; 50 Department of Molecular Biology and Biochemistry, Indiana University School of Medicine, Indiana University-Purdue University Indianapolis, 635 Barnhill Drive, Indianapolis, IN 46202, USA; 51 Department of Immunity and Infection, School of Medical and Dental Sciences, University of Birmingham, Wolfson Drive, Edgbaston, Birmingham, West Midlands B15 2TT, UK; 52 Immunobiology and Cancer Program, Oklahoma Medical Research Foundation, 825 NE 13th Street, Oklahoma City, Oklahoma 73104, USA; 53 Radiation Oncology, Department of Pharmacology and Experimental Therapeutics, Jefferson University Hospital, 1020 Locust Street, Philadelphia, PA 19107, USA; 54 Department of Microbiology and Immunology, University of Oklahoma Health Sciences Center, University of Oklahoma, 100 North Lindsay Avenue, Oklahoma City, OK 73104, USA; 55 Department of Biology, McMaster University, LSB-331, 1280 Main Street West, Hamilton, Ontario L8S4K1, Canada; 56 School of Medicine, Johns Hopkins University, 720 Rutland Avenue, Baltimore, MD 21205, USA; 57 Department of Pharmacology and Experimental Therapeutics, Jefferson Medical College, Thomas Jefferson University, 132 South 10th Street, 1170 Main, Philadelphia, PA 19107, USA; 58 Discipline of Biochemistry, School of Molecular and Biomedical Science, University of Adelaide, North Terrace, Adelaide, South Australia 5005, Australia; 59 Institute of Cellular Medicine, Faculty of Medicine, Newcastle University, Medical School, Framlington Place, Newcastle upon Tyne, Tyne and Wear NE1 7RU, UK; 60 Department of Immunity and Immunology, School of Medical and Dental Sciences, University of Birmingham, Wolfson Drive, Edgbaston, Birmingham, West Midlands B15 2TT, UK; 61 Department of Cell Biology and Histology, Center for Immunology Amsterdam, Academic Medical Center, University of Amsterdam, Meibergdreef 15, Amsterdam, Noord Holland 1105 AZ, The Netherlands; 62 Division of Cancer Imaging Research, Department of Radiology, School of Medicine, Johns Hopkins University, 720 Rutland Avenue, Baltimore, MD 21205, USA; 63 Genome Center, University of California at Davis, 1 Shields Avenue, Davis, CA 95616, USA; 64 University of California at San Diego, 9500 Gilman Drive, San Diego, CA 92093, USA; 65 Department of Biochemistry and Developmental Genomics Group, Center of Excellence in Bioinformatics and Life Sciences, State University of New York at Buffalo, 701 Ellicott Street B3-303, Buffalo, New York 14203, USA; 66 Department of Pathology and Molecular Medicine, Queen's Cancer Research Institute, Queen's University, 18 Stuart Street, Botterell Hall, Kingston, Ontario K7K 4G4, Canada; 67 Department of Molecular and Cellular Biology, Department of Laboratory Medicine and Pathobiology, Sunnybrook Health Sciences Centre, University of Toronto, 2075 Bayview Avenue, Toronto, Ontario M4N 3M5, Canada; 68 Department of Molecular and Cellular Biology, Sunnybrook Health Sciences Centre, University of Toronto, 2075 Bayview Avenue, Toronto, Ontario M4N 3M5, Canada; 69 Institute of Cellular Medicine, Newcastle University, Medical School, Framlington Place, Newcastle upon Tyne, Tyne and Wear NE2 4HH, UK; 70 Faculty of Medical Sciences, Institute of Cellular Medicine, Newcastle University, Medical School, Framlington Place, Newcastle upon Tyne, Tyne and Wear NE2 4AA, UK; 71 Department of Pathology and Laboratory Medicine, David Geffen School of Medicine, University of California at Los Angeles, 10833 Le Conte Avenue, Los Angeles, CA 90095-1732, USA; 72 Radiology and Oncology, School of Medicine, Johns Hopkins University, 720 Rutland Avenue, Baltimore, MD 21205, USA; 73 Department of Environmental Health Sciences, Johns Hopkins Bloomberg School of Public Health, Johns Hopkins University, 615 North Wolfe Street, Baltimore, MD 21205, USA; 74 Department of Pharmacology and Toxicology, Neuroscience Institute, Morehouse School of Medicine, 720 Westview Drive Southwest, Atlanta, GA 30310, USA; 75 Department of Pharmacology, Perelman School of Medicine, University of Pennsylvania, 10-124 Translational Research Center, 3400 Civic Center Boulevard Building 421, Philadelphia, PA 19104-5158, USA; 76 Department of Neurological Surgery, Seattle Children's Research Institute, University of Washington, 1900 Ninth Avenue, Seattle, WA 98101, USA; 77 Faculty of Biology and Medicine, Center for Integrated Genomics, University of Lausanne, CH-1015 Lausanne, Lausanne, Vaud CH-1015, Switzerland; 78 Department of Biomedical Sciences and Pathobiology, VA-MD Regional College of Veterinary Medicine, Virginia Polytechnic Institute and State University, Duck Pond Drive, Blacksburg, VA 24061, USA; 79 Department of Molecular Genetics and Cell Biology, University of Illinois at Chicago, 920 East 58th Street, Chicago, IL 60637, USA; 80 Center for Integrative Brain Research, Seattle Children's Research Institute, University of Washington, 1900 Ninth Avenue, Seattle, WA 98101, USA; 81 Clinical Endocrinology Branch, National Institute of Diabetes, Digestive, and Kidney Disorders, National Institutes of Health, 10 Center Drive, Bethesda, MD 20892-1772, USA; 82 Department of Biosciences and Nutrition, Novum, Karolinska Institutet, Hälsovägen 7-9, Huddinge, Stockholm SE-141 83, Sweden; 83 Department of Biochemistry, University of Buffalo School of Medicine and Biomedical Sciences, State University of New York at Buffalo, 701 Ellicott Street, Buffalo, NY 14203, USA

## Abstract

Here we present the Transcription Factor Encyclopedia (TFe), a new web-based compendium of mini review articles on transcription factors (TFs) that is founded on the principles of open access and collaboration. Our consortium of over 100 researchers has collectively contributed over 130 mini review articles on pertinent human, mouse and rat TFs. Notable features of the TFe website include a high-quality PDF generator and web API for programmatic data retrieval. TFe aims to rapidly educate scientists about the TFs they encounter through the delivery of succinct summaries written and vetted by experts in the field. TFe is available at http://www.cisreg.ca/tfe.

## Background

**Figure 1:**
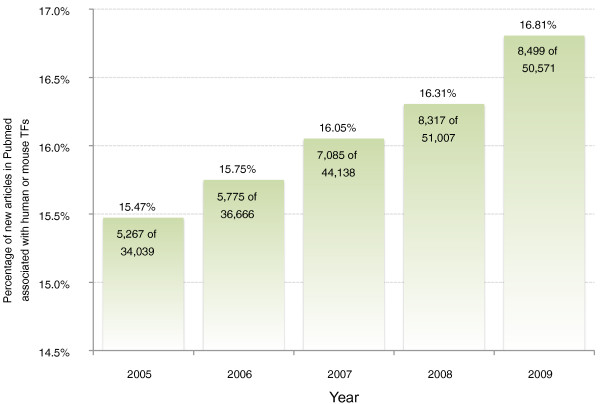
**New journal articles associated with human or mouse TFs**. Over the past five years, 216,421 journal articles associated with human or mouse genes have been published and indexed in NCBI PubMed. This amount represents 5.59% of all articles published and indexed during the same time frame (3,871,190 articles). Out of the 216,421 articles associated with human or mouse genes, at least 34,943 are associated with human or mouse TFs, or 16.15%. This is astounding when considering that known TFs represent only 5% of the genome. The proportion of journal articles associated with TFs has also been rising steadily over the past five years - from 15.47% in 2005 to 16.81% in 2009. These figures were determined with a conservative set of approximately 3,200 human and mouse TF genes derived from the works of Fulton *et al*. [4] and Vaquerizas *et al*. [5] and the publicly available 'gene2pubmed' annotation from NCBI.

As modulators of gene expression, transcription factors (TFs) act on all eukaryotic biochemical systems, driving 'networks' or 'regulatory programs' that define the developmental stages of life and maintain cells in dynamically changing microenvironments. From regulating muscle differentiation in embryonic development (*MYOD*) [1] to helping the kidneys reclaim water at times of dehydration (*NR3C2*) [2] and even instigate oncogenesis (*MYC*) [3], the pervasive roles of TFs are becoming increasingly appreciated and experimentally characterized. TFs are amongst the most highly studied class of proteins. Even though TFs comprise fewer than 5% of human protein-encoding genes [4,5], over 16% of gene-related papers address members of this critical class (Figure 1).

Increasingly, TFs are the focus of research aimed at deciphering the complex regulatory programs that allow a single genome to specify hundreds of phenotypically distinct cell types. The study of stem cell differentiation is dominated by efforts to understand how the activation of individual TFs can direct the progression to specific lineages. Perhaps the most important of these advances in recent years is the realization that, by introducing specific 'sets' of TFs into terminally differentiated cells, one can induce these cells to return to a pluripotent capacity [6,7]. A complete understanding of TFs and the processes that alter their activity is a fundamental goal of modern life science research.

Rapidly advancing knowledge in TFs is nearly impossible to track, with over 8,000 TF-related papers published in 2009 alone (Figure 1). In this light, the authors of this work believe that non-TF researchers are sometimes confronted with the need to understand the properties of certain TFs that they come across within their research, as a potential participant in some differentiation, signaling or regulatory pathway they are studying. In this scenario, an accessible, high quality synopsis of the TF can catalyze rapid progress in the study, allowing researchers to chart an efficient approach. Such synopses have traditionally been obtained from published review articles, but the need for timely information about the growing pool of actively studied genes has increasingly led researchers to online information sources.

In the Internet Age, gene-specific resources have emerged that present information gathered from highly specialized biomedical databases. Examples of such resources include Entrez Gene [8] and GeneCards [9]. While automated content can be useful, many researchers seek summary descriptions of the proteins. The classic UniProt/Swiss-Prot [10,11] model for curated content is often viewed as a gold standard, while automated systems have emerged to extract key sentences from the research literature, such as iHOP [12] and WikiGenes [13]. The community participation model for maintaining current information exemplified by Wikipedia has arguably not been proven successful for small communities with specialized interests and need for peer-reviewed content, perhaps reflecting the limited time available from the small cadre of qualified experts. The Gene Wiki project within Wikipedia has been the most advanced effort, providing automated stub articles for many genes within the confines of Wikipedia [14]. However, the absence of a rigorous and enforced peer review process and the lack of oversight in monitoring contributor qualifications makes the model less than ideal for scientists who seek *bona fide *information in the digital realm.

TFs are proteins with special abilities and attributes not found in other classes of proteins. For example, they often work in pairs or networks to modulate specific regulatory pathways. They directly or indirectly bind to DNA. Some also interact with ligands or hormones. In short, the unique properties of TFs place special demands on - and presents opportunities for innovation with regards to - the kind of information TF-specific biomedical resources can offer, and how this information can be displayed to users such that it is intuitive, sensible, and helpful. There are many different kinds of TF-specific useful data that can be captured. Sequence-specific DNA binding TFs act on target genes, interact with other TFs to achieve specificity in action, and have structural characteristics that are predictive of DNA interaction mechanisms. A well-characterized TF will be represented by a binding profile that defines the target sequences to which it can bind. These class-specific properties have spurred the development of key databases, such as JASPAR [15], PAZAR [16] and TRANSFAC^® ^[17]. These efforts, however, are constrained by limited capacity to identify and curate data from the scientific literature.

**Figure 2:**
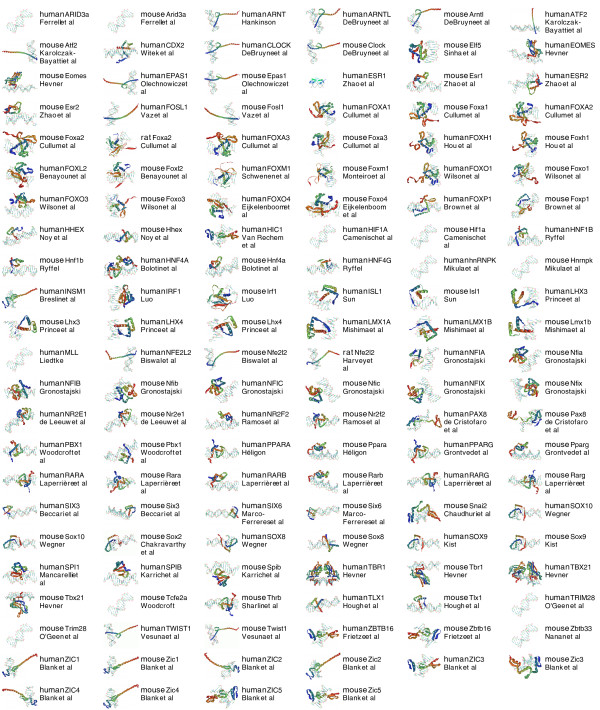
**Released mini review articles**. These mini review articles - listed in alphabetical order - are those that have been sufficiently completed and released by their respective authors. These articles can be accessed at [43].

**Figure 3:**
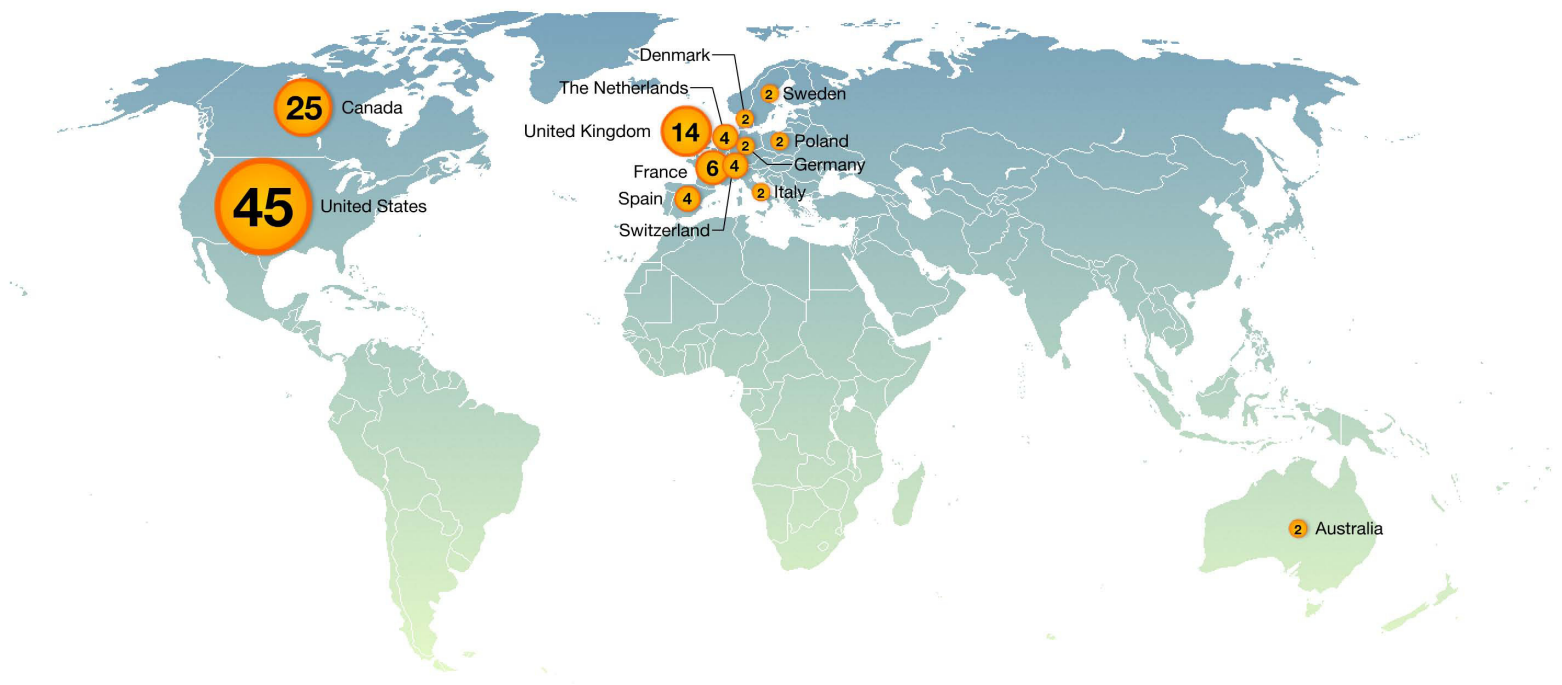
**Worldwide distribution of authors by country**. The TFe consortium comprises 114 authors from 13 countries. The exact distribution is as follows: Australia, 2; Canada, 25; Denmark, 2; France, 6; Germany, 2; Italy, 2; Poland, 2; Spain, 4; Sweden, 2; Switzerland, 4; the Netherlands, 4; the United Kingdom, 14; and the United States, 45.

Based on the importance of TFs, the rapid accumulation of research advances in the scientific literature, and the need to provide class-specific information, we have created a new web-based platform called the Transcription Factor Encyclopedia (TFe). TFe's mission is to facilitate the curation, evaluation, and dissemination of TF data. TFe espouses the principles of open access and promotes collaboration within the TF research community. It rewards scientists for contributing their data, and aims to optimize content quality ensuring expert editorship and multiple levels of peer review, both internal and external. TFe is curated and managed by the TFe consortium, a collaboration of over 100 TF researchers from throughout the world (see Figure 2 for the list of completed mini review articles that they contributed, and Figure 3 for their distribution by country). The objective of the TFe consortium is to produce concise mini review articles on pertinent human and mouse TFs, and to accelerate the curation of TF-specific data.

To date, the TFe consortium has prepared over 800 TF mini review articles, 136 of which are sufficiently complete to be presented here in the inaugural paper. Overall, the TFe database contains 184 original tables and diagrams, 221 TF binding site profiles, 3,083 non-redundant binding sequences, 2,334 genomic targets, 212 three-dimensional structural predictions, 6,308 protein-protein and protein-ligand interactions, 42,500 TF-to-disease predictions based on Medical Subject Headings (MeSH), and more.

The long-term goal of TFe is to create an online encyclopedic collection about well-studied TFs, combining a mixture of both expert-curated and automatically populated content.

## Resource content

In this paper we present a collection of 136 mini review articles about human and mouse TFs. These articles are available on the TFe website [18]. Two versions of every article are available. A definitive version can be viewed online, while an abridged version can be downloaded in Portable Document Format (PDF) from the website. A sample PDF article is enclosed in Additional file 1, while Additional file 2 contains the raw data files.

The completed articles represent 15% of all TF articles that have been pre-populated with automated content in TFe. As for the remaining articles, most are awaiting an expert volunteer author or remain at a preliminary state of development. An ongoing effort aims to recruit appropriate authors to curate these 'orphaned' articles. In total, TFe currently hosts 803 TF articles, 216 of which are human, 585 of which are mouse, and 2 of which are rat. While TFs that bind directly to DNA are considered for inclusion in TFe at this time, a few contributed articles have addressed other TFs. Recent research has suggested that there are well over 1,300 TFs in the human genome [4,5]. With the increasing availability of data, our goal is to eventually characterize all TFs in the human and mouse genomes. See Additional file 3 for an inventory of all TF articles currently available in TFe alongside their classification, which is discussed in further detail below.

### Article structure

To ensure uniformity, all TF articles in TFe are written in a standardized format that was established in response to input and feedback from consortium members. The style emphasizes relatively short articles - accompanied by a few figures and up to 75 references. These articles are concise, informative, and cater to a broad audience of life science researchers.

**Figure 4:**
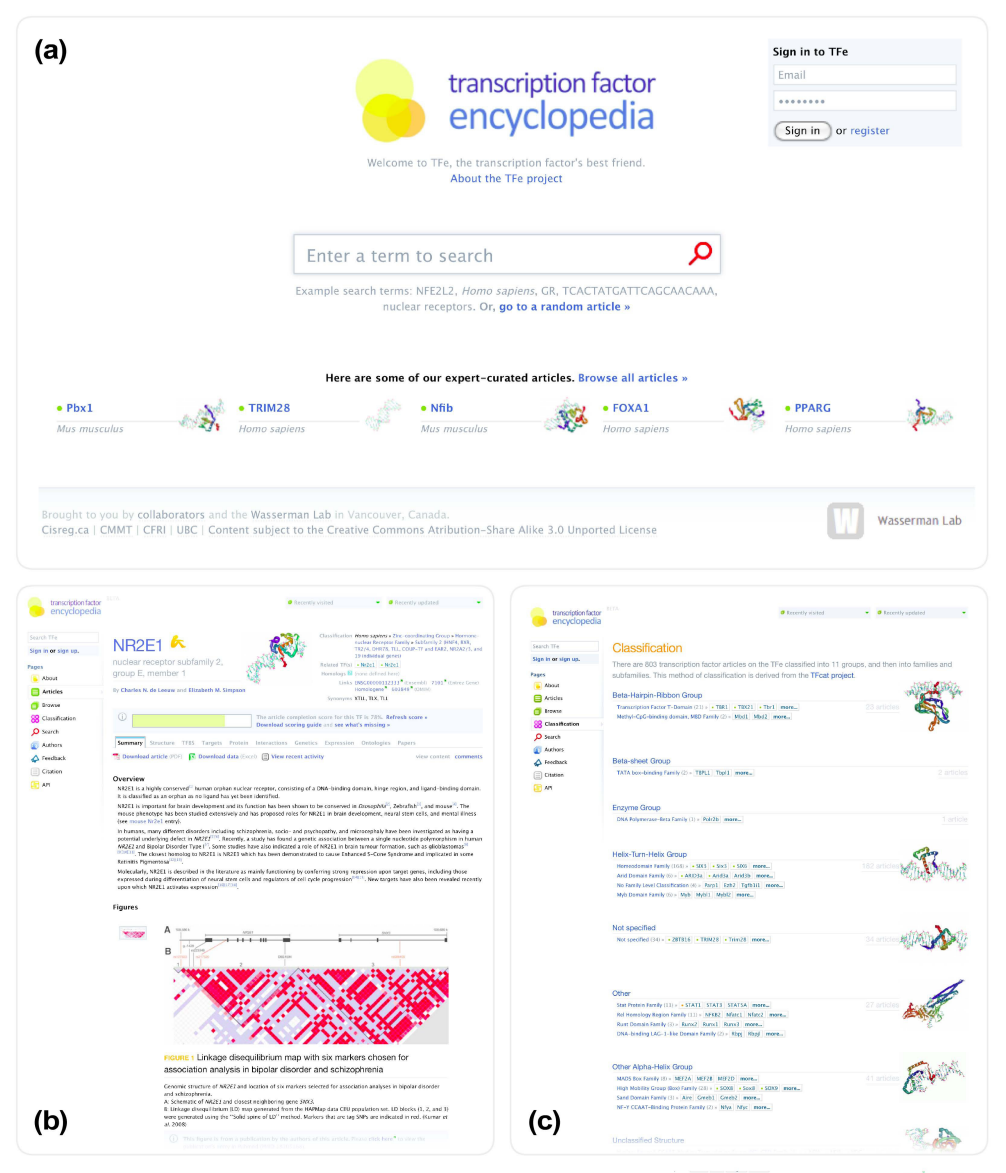
**TFe user interface**. Shown in this figure are three screenshots from the TFe web-based user interface. Built around HTML, JavaScript and CSS standards, the TFe user interface is a quick and powerful method of viewing, downloading, and editing TFe data. Pages visualized in this figure are: (a) the home page; (b) the article page; and (c) the classification page.

**Figure 5:**
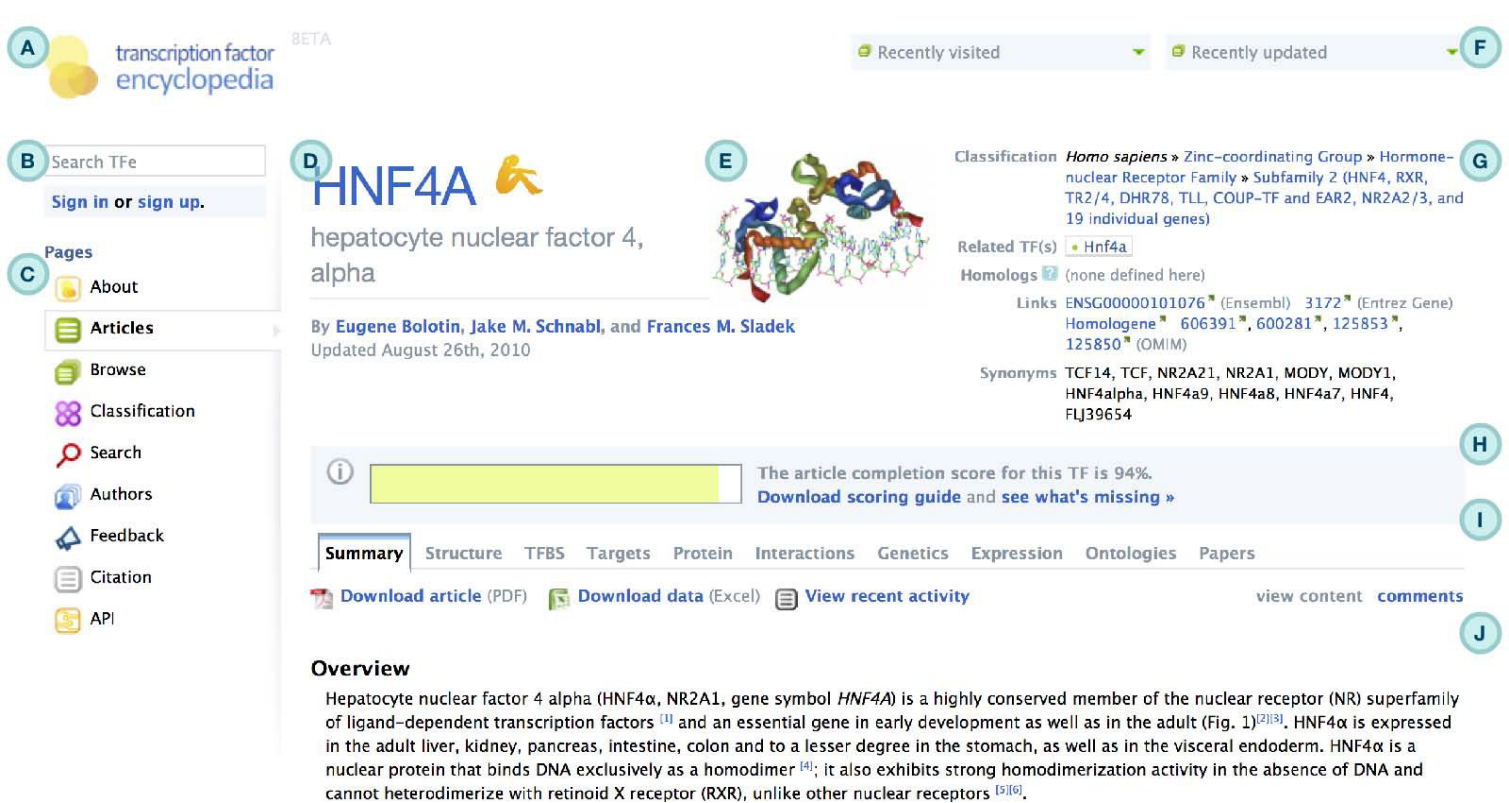
**Tour of the user interface**. (A) The project logo links back to the homepage. (B) The 'quick search' and 'sign in' widgets are conveniently placed near the top of the page. (C) The vertical site navigation bar offers fast access to all available pages in the site. (D) The official symbol, name, and authors are prominently placed to immediately grab the user's attention. Beneath the authors' names is the date of the most recent revision. (E) When available, a thumbnail of the structural prediction rendering is displayed in the header area. (F) Two drop-down menus provide easy access to the top ten most recently visited and updated articles. (G) Vital information on the TF, such as its classification, homologs, genomic links, and synonyms, occupy the top right corner of each page. (H) An article completion score bar provides immediate feedback to the author on the progress of their articles. (I) Articles in TFe are organized into ten tabs. Immediately underneath, the tabs are links to data downloads in PDF and Excel file formats. A 'view content, comments' toggle allows the user to view comments that have been attached to the article. By default, comments are hidden from sight. (J) Most tabbed sections start with an author-contributed 'summary' paragraph that ranges in length from 150 to 500 words.

**Figure 6:**
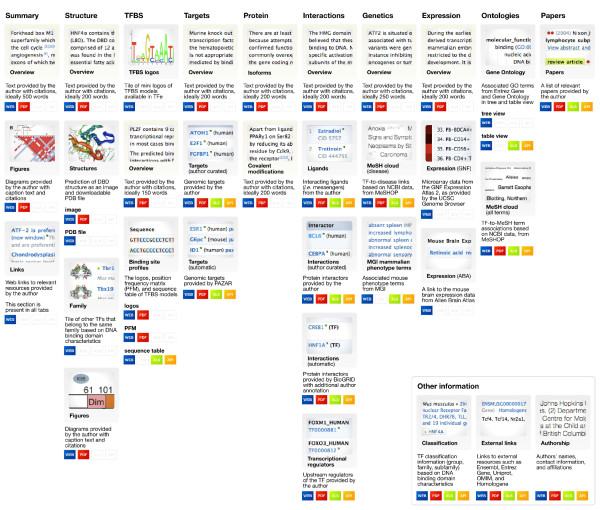
**Content available in TFe**. This diagram demonstrates the diverse range of TF-related content available in TFe. Articles in TFe are organized into ten tabs. In this diagram, the ten tabs are represented by the ten horizontal columns labeled 'Summary', 'Structure', 'TFBS', and so forth. Under each tab in the article, there exist one or more relevant subheadings. In this diagram, these subheadings are represented by beige or grey boxes, which contain partial screenshots of the actual content - whether they are text, figures, or tables. Beige boxes represent content that has been composed by TFe authors, while grey boxes represent content that has been largely automatically populated. Below each screenshot box is the name of the subheading and a brief description of the subheading. Below the description are a series of blue, red, green, and yellow icons labeled 'WEB', 'PDF', 'XLS', and 'API'. As the names suggest, these icons indicate whether the content of that particular subheading is available in various formats. All subheadings are available in web format - on the TFe website. Thus, we consider the TFe website format as the most comprehensive format available. Select content is available in redacted form in the PDF format. Content that is in the form of 'data' can be downloaded as an Excel spreadsheet ('XLS') or retrieved using the TFe web API ('API') from the TFe website.

The article page (shown in Figure 4b) is the cornerstone of the TFe website, as it is where articles are accessed. Articles in TFe are organized into ten tabbed sections titled 'Summary', 'Structure', 'TFBS' (TF binding site), 'Targets', 'Protein', 'Interactions', 'Genetics', 'Expression', 'Ontologies', and 'Papers' (that is, references) (Figure 5). Above the tabs lies a standard header that displays pertinent information regarding the TF, including the TF symbol, species, classification, the date of the most recent revision, and an article completion score bar (Figure 5). Sections generally contain a mixture of author-curated and automatically populated content, typically in the form of an expert-written overview text - the author-curated portion - followed by several additional headings filled with a mixture of author-curated and automatically populated content. See Figure 6 for a comprehensive list of all automatically populated and manually curated content available in the article page. The automatically populated content represents data that we have incorporated into TFe from second and third party resources, including: BioGRID [19], Ensembl [20], Entrez Gene [8], Gene Ontology [21], MeSH [22], the Mouse Genome Database [23], Online Mendelian Inheritance in Man (OMIM) [24], PAZAR [25], RCSB Protein Data Bank [26], the UCSC Genome Browser [27] and the Allen Brain Atlas [28]. More details on the software tools and data repositories utilized in the generation of automatically populated content found in each tab are presented in Table 1.

**Table 1:** Sources of automatically populated content

Tab	Section	Sources	Use of sources
Structure	Structures	RSCB PDB, Pfam	Structural predictions are made with the help of experimentally verified protein structures downloaded from the RSCB PDB. In the process of creating the structural predictions, we use the HMM database from Protein Families (Pfam) to help us identify domains found in protein sequences in the RSCB PDB database (which we use as templates) as well as the protein sequences of putative structures we want to predict
TFBS	TFBS logos	PAZAR	The logo in this section is generated with the Perl module MEME and its dependencies, using binding site data from PAZAR
	Binding site profiles	PAZAR	The logo and position frequency matrix in this section are generated with the Perl module MEME and its dependencies, using binding site data from PAZAR
Targets	Targets (author curated)	Gene Ontology (NCBI)	While the author provides the gene ID, TF complex, effect, and reference, biological process GO terms associated with each target gene in this section are imported from gene-to-GO annotations from NCBI
	Targets (automatically populated)	PAZAR, Gene Ontology (NCBI)	Target gene, TF complex, and reference data are imported from PAZAR. The author supplies effect data. Biological process GO terms associated with each target gene in this section are imported from GO annotations provided by NCBI
Interactions	Ligands (author curated)	PubChem (NCBI)	While ligand IDs, experiment types, natures of interaction and references are supplied by the author, the ligand common name and image are provided from PubChem
	Interactions (automatically populated)	BioGRID	Interactor names, experiment types, and references are imported from BioGRID. Natures of interaction are provided by the author
	Transcriptional regulators (automatically populated)	PAZAR, Gene Ontology (NCBI)	Regulating TF complex, regulating TF, genomic links, and reference information are provided by PAZAR. Biological process GO terms associated with each target regulator in this section are imported from GO annotations provided by NCBI
Genetics	MeSH cloud (automatically populated)	MeSH (NCBI), Entrez Gene, GeneRIF	MeSH term associations and Fisher's exact *P*-values are generated using data from NCBI MeSH, Entrez Gene, and GeneRIF
Expression	Expression (automatically populated)	UCSC Genome Browser, Allen Brain Atlas	Expression data in this section are imported from the UCSC Genome Browser database, GNF Expression Atlas 2 dataset, and the Allen Brain Atlas
Ontologies	Gene Ontology (automatically populated)	Gene Ontology (NCBI)	GO terms associated with the transcription factor in this section are imported from GO annotations provided by NCBI
	MeSH cloud (automatically populated)	MeSH (NCBI), Entrez Gene, GeneRIF	MeSH term associations and Fisher's exact *P*-values are generated using data from NCBI MeSH, Entrez Gene, and GeneRIF
Papers	Papers	PubMed	Detailed information on relevant papers such as authors, titles, journals, and publication dates are imported from NCBI PubMed.

Eight out of ten tabs in TFe articles contain one or more subheadings of automatically populated content. This table lists the direct sources of automatically populated content by tab and subheading ('section'). GO, Gene Ontology; RSCB, Research Collaboratory for Structural Bioinformatics.

Each section - with the exceptions of the 'Ontologies' and 'Papers' sections - begins with a brief, expert-written summary statement from the authors followed by relevant figures, lists, and tables. For instance, the 'Summary' section is designed to begin with a 500-word (maximum) overview followed by one or two captioned figures. The 'Targets' section contains a 200-word overview focusing on the TF's regulatory role, followed by a table of genomic targets populated by the author and additional data automatically extracted from PAZAR. The expert-written summaries in TFe are meant to provide the reader with some perspective, highlight key points, and reveal tacit knowledge. For a complete list of features available in each section, please see Additional file 4.

Here we discuss each of the ten tabbed sections - 'Summary', 'Structure', 'TFBS', 'Targets', 'Protein', 'Interactions', 'Genetics', 'Expression', 'Ontologies', and 'Papers' - in greater detail.

#### Summary tab

The 'Summary' tab presents insightful overview text written by expert authors, one or more figures as supplied by them, and a list of relevant references. Authors also have the option to post noteworthy links - for instance, to a Wikipedia entry for the TF.

Like every other tab, the 'Summary' tab user interface is a content viewer and editor combined into one. When expert authors wish to implement changes to their articles, they may 'sign in' to TFe using their personalized user accounts. After this is done, they are able to see the normally hidden editing interface that allows them to upload text, figures, figure captions, references, external links, and data, depending on the tab. The editing interface supports the widely used wiki syntax to allow basic text formatting, such as bolding, italicizing, underlining, and the creation of bulleted and numbered lists. All text entered in wiki syntax is converted to HTML by a local installation of the MediaWiki software. Authors also have the option to add PubMed references anywhere in their text by using special tags that look like '(pmid:16371163)' - without the quotes. These tags are automatically converted to a proper citation (Vancouver style) by the TFe software. Figures can be uploaded in many different image formats, while figure captions are submitted as text. PubMed citations are also supported in figure caption text.

#### Structure tab

The 'Structure' tab contains author-provided overview text regarding the structural properties of the TF, followed by - if available - the predicted three-dimensional structure of the TF's DNA binding domain. These 'structural predictions', which were created by the consortium using a custom-made pipeline, are available for download as both high-resolution Portable Network Graphics (PNG) images and Protein Data Bank (PDB) formatted files. The materials and methods used in their construction are discussed in the Materials and methods section of this paper.

#### TFBS tab

A key property of TFs is the DNA sequences to which they bind. In the world of TF research, such DNA sequences are often called 'transcription factor binding sites', or 'TFBS' for short. Knowledge of TFBS patterns is key to identifying putative binding sites in genomic sequences and to the identification of sets of genes regulated by the TF in promoter analysis.

In light of this, disseminating TFBS data is a crucial part of TFe's mission. The 'TFBS' tab contains a summary of the DNA binding characteristics of the TF, alongside one or more DNA binding target site data, when sufficient data are available. A graphical depiction of the target site pattern is displayed in the form of a sequence logo, along with a brief summary text from the author. This information is extracted from the PAZAR regulatory sequence database.

TFe authors are able to create new binding models by inputting a list of binding sites, experimental evidence, and references in the 'TFBS' tab through a submission interface that is visible to authors only. It is possible for authors to submit target sequences that exist in a genome, or artificial sites, such as those generated in a SELEX (Systematic Evolution of Ligands by Exponential Enrichment) experiment. When we receive a submission through this TFBS form system, we forward the supplied information to a team of curators who review the information for errors and, if appropriate, deposit the annotation into the PAZAR database. Because PAZAR and TFe are programmatically linked, the annotation deposited in PAZAR will also appear in TFe.

#### Targets tab

Related to the 'TFBS' tab, the 'Targets' tab presents users with an introductory text followed by a list of genes directly regulated by the subject TF sourced from the PAZAR database. At a minimum, the 'Targets' list recapitulates the information in the 'TFBS' tab, but oftentimes, expert authors provide additional genes known to be regulated by the TF but for which the specific DNA target sequence is unknown. Authors can add additional targets by using a specialized editing interface that is accessible upon sign in.

#### Protein tab

The 'Protein' tab presents information about the functional consequences of protein modifications or distinctions between protein isoforms. Authors summarize such information in free text entries. As a late addition to the system identified as a need during the beta-testing process, the section has yet to be populated for many entries.

#### Interactions tab

Interactions between TFs and ligands or proteins are reported in this tab. While automated content from the BioGRID database is included, authors may also provide information about additional interactions not reported in the external system through a specialized submission interface. Authors have a limited set of interaction types (Table 2) from which to pick labels. If the gene encoding the TF is subject to transcriptional regulation in a selective manner, the regulating TFs are reported in this section.

**Table 2:** List of predefined interaction types

Interaction type	Gene	Ligand
Acts on upstream signaling pathway		•
Competitive inhibition	•	•
Genetic	•	•
Indirect	•	•
Multimerization	•	•
Not specified	•	•
Physical: deacetylation	•	•
Physical: dephosphorylation	•	•
Physical: desumoylation	•	•
Physical: deubiqiutination	•	•
Physical: enzyme modification: acetylation	•	•
Physical: enzyme modification: methylation	•	•
Physical: enzyme modification: phosphorylation	•	•
Physical: enzyme modification: protein cleavage	•	•
Physical: enzyme modification: sumoylation	•	•
Physical: indirect altering posttranslational modifications	•	•
Physical: sequestering	•	•
Physical: translocation	•	•
Physical: ubiquitination	•	•
Physical: undefined direct interaction	•	•
Physical: with another TF	•	
Physical: with another TF: complex binds DNA	•	
Physical: with co-activator affecting recruitment	•	•
Physical: with co-repressor affecting recruitment	•	•
Regulatory: decreases expression of this TF	•	•
Regulatory: increases expression of this TF	•	•
Unknown	•	•

When a protein-to-protein or protein-to-ligand is added to a TFe article either by the author or automatically from BioGRID, the author of that article has the option to define the interaction type if this information is known. To promote a standardized vocabulary for describing interaction types, a list of possible interaction types between proteins and ligands is provided to the author. This list continues to be adjusted and expanded based on need and author feedback.

#### Genetics tab

TFs perform powerful genetic roles in the development and physiology of organisms. Therefore, the genetic properties of TFs can have powerful consequences upon the phenotype of an organism. The 'Genetics' tab presents two sets of data linking TFs to phenotype, in addition to the prerequisite expert-written summary. The first is a 'cloud' of TF-to-disease associations composed with MeSH terms. The second set of data linking TFs to phenotype is a list of Mouse Genome Database mammalian phenotype terms associated with the mouse homolog of the TF protein.

#### Expression tab

The 'Expression' tab reports expression data from the GNF Expression Atlas, sourced from the UCSC Genome Browser, and observed regional expression in the brain according to the Allen Brain Atlas. Authors are encouraged to provide a text description of known expression properties of the TF gene.

#### Ontologies tab

Annotated characteristics of the TF are reported in the 'Ontologies' tab. Gene Ontology terms linked to the gene are extracted from Entrez Gene for display. The automatically populated TF-to-MeSH associations for all MeSH terms outside of diseases are reported, following the same procedure as introduced in MeSHOP.

#### Papers tab

The 'Papers' tab provides a set of recommended articles pertinent to the TF. Authors indicate the most useful introductory readings and other key papers with a two circle rating system. Two full circles indicate an excellent paper in the author's opinion, while no circles still indicate a very good and noteworthy paper.

## System features

In this section of the paper, we discuss the important features of our platform. These features include: (1) our system of classifying TFs; (2) our concept of 'content inheritance', or how articles of very closely related TFs may derive content from each other when biologically appropriate; (3) our structural prediction system; (4) our data on TF binding sites; (5) our TF-to-disease association predictions; (6) our PDF rendering system; (7) TFe's data export capabilities; and (8) the article completion score.

### Classification of transcription factors

The classification of TFs into 'groups', 'families' and 'subfamilies' is a very important feature of TFe. Over the past few years, there have been efforts to identify and classify all TFs within the human and mouse genomes [4,5]. While there are potentially several different strategies for classifying TFs, one promising approach is to group them based on DNA binding domain structures. Building upon the work of Fulton *et al*. in the Transcription Factor Catalog (TFCat) project [4], we have organized all TFs in TFe into various groups, families and subfamilies as previously mentioned (Additional file 3).

### Content inheritance

When comparing orthologous TFs, or recently evolved paralogs within a species, it is commonly observed that homologous TFs are well conserved structurally and functionally [29]. Indeed, some homologous TFs are so well conserved that there is often no information that distinguishes the homologs. However, in TFe we have opted to create separate articles for all TFs, including homologous TFs. For instance, we have several articles for the TF NFE2L2 - one each for human, mouse, and rat. In doing so, we aim to provide maximum flexibility to our authors who may wish to discuss key subtle differences between closely related proteins.

The drawback to this approach is that, in some cases, we end up with multiple articles for what could be considered as functionally synonymous TFs. These TFs, due to their extreme likeness, would inevitably share common attributes such as binding site profiles, interactors, and target genes. In this situation, it becomes important to keep all shared attributes current and synchronized across the different articles. To assist with this information management process, we implemented a content inheritance system that enables authors to define small clusters of homologous TFs for which certain data may be automatically shared. Under this system, the article that is more annotated - the 'parent article' - donates text, figures, and data as appropriate to the article that is less annotated, the 'child article'. However, authors and editors are able to override the automatic sharing of data when it is not reflective of the underlying biology.

### Structural predictions

**Figure 7:**
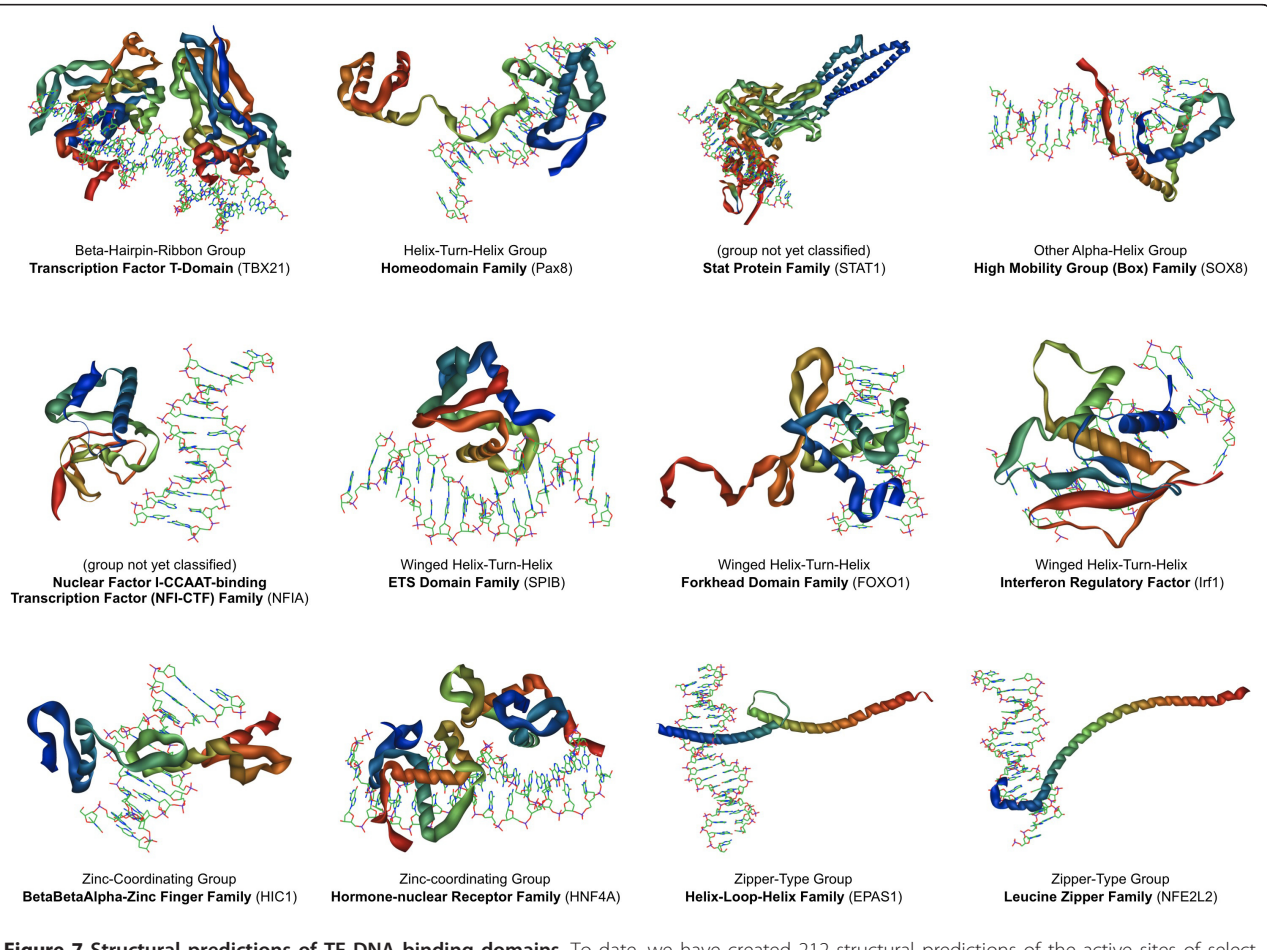
**Structural predictions of TF DNA binding domains**. To date, we have created 212 structural predictions of the active sites of select TFs in TFe. We focused on TFs for which a structural prediction is most feasible and whose articles are nearing completion. These predictions were generated with an in-house, custom-made pipeline that finds the most similar, experimentally determined protein structure for each unsolved TF, and uses that experimentally determined 'template' to guide the prediction of the unknown structure.

We have developed a custom computational pipeline for predicting the three-dimensional protein structures of the DNA binding domains of TFs. The final output of our pipeline is a PDB formatted file of the predicted structure, alongside a short segment of double-stranded DNA for positional reference. The DNA molecules are stylistic and do not represent particular sequences such as the consensus sequence for the TF. We have generated standardized PNG image renderings of these PDB files for web and print purposes. Figure 7 contains a representative sample of the structural predictions, one from each family of TFs featured in our first release. To date, 212 structural predictions have been generated, with the emphasis of effort focused on TFs with articles that are nearing completion. All structural predictions are available for download in PDB format under the 'Structure' section in the articles of their respective TFs. A brief summary of the materials and methods used in our protocol can be found in the Materials and methods section below.

### Transcription factor binding site data

One of the goals of TFe is to encourage experts to assist in the curation of TFBS sequences and generation of binding profiles. Working in partnership with PAZAR [25], an open source and open access TF and regulatory sequence annotation database, our consortium gains access to a powerful curation platform with which it can store, annotate, and manage data, as well as retrieve additional data from other projects in PAZAR. Our initial collection of 100 reviews collectively contain 3,083 unique binding site sequences from the PAZAR database, of which a total of 452 sequences have been donated to PAZAR by the consortium. From this set of binding site sequences, we have generated 221 binding models and extracted 1,436 genomic targets for 199 different TFs. In addition, 898 genomic targets have been entered manually by our authors to supplement this genomic target dataset. See Additional file 2 to see the binding data of released articles and Additional file 5 for key binding profiles that have been generated in the TFe project.

### Disease associations

Many TFs are implicated in disease. Out of a growing list of 1,321 human TFs we compiled from the work of Vaquerizas *et al*. [5] and Fulton *et al*. [4], 197 are currently linked to one or more diseases in the OMIM Morbid Map [30]. In light of the strong connection between TFs and disease, we have predicted 42,500 TF-to-disease associations. This was done by using the 'Entrez Gene to PubMed' ('gene2pubmed') and MeSH datasets that are available at the National Center for Biotechnology Information (NCBI). With mainly these two datasets, with additional datasets such as OMIM and GeneRIF to further strengthen our predictions, we developed a protocol that makes the connection between TF-encoding genes, papers that discuss these genes, and the MeSH terms that are tagged to the papers. By indirectly mapping disease-oriented MeSH terms to TF-encoding gene identifiers, we are able generate a list of MeSH terms that are associated with each TF. Statistical analysis is applied to the raw connections to determine their strength - mainly by reflecting the frequency of TF-term co-occurrence in light of the number of papers that refer to either the TF or the term. This information can be viewed as a table or as a 'cloud' under the 'Genetics' tab.

### PDF rendering

**Figure 8:**
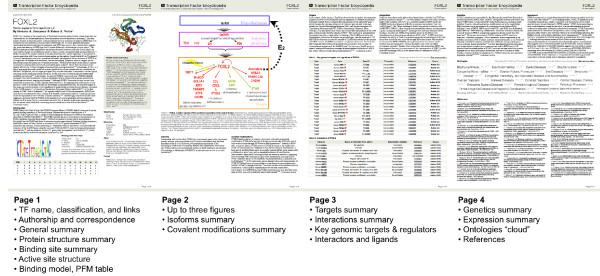
**Format of the PDF article**. The PDF mini summaries are composed of four pages. The first page features basic information such as the TF name, gene identifiers and classification, as well as author information. Also on the first page are the names and affiliations of the authors, an overview of the TF, an image of its active site protein structure accompanied by a brief commentary, and a featured TF binding profile selected by the author. The second and third pages contain a mixture of figures, paragraph text, and tables of genomic targets and protein as well as ligand interactors. The last page contains two brief paragraphs, a MeSH cloud, and selected references. These are the first two pages of a four-page PDF mini summary generated by the TFe system software. Our PDF creation tool, based on in-house code and the dompdf 0.5.1 open source module, is able to format a TFe article of any length and annotation depth as a standardized four-page PDF article. A fuzzy logic algorithm does all of the modifications necessary to make the conversion. These modifications may include changing the sizes of the figures, truncating excess text, reformatting the references, and calculating trade-offs between having larger figures and data tables at the expense of less text, or keeping more text at the expense of having fewer figures and smaller data tables.

We have built a PDF rendering engine in TFe that transforms articles into condensed, four-page PDF 'mini summaries' available for printing (Figure 8). These summaries can be downloaded by clicking on the 'Download article (PDF)' link that is prominently displayed on all article pages on the TFe website. We have included a sample in Additional file 1.

While the articles as they appear on the TFe website permit great flexibility in terms of length and variety of content, the PDF format is more structured and compact. Thus, the PDF version of the articles can be described as the 'abridged' form of the article. When necessary, we are keen to remind users that there is additional content on the TFe website that cannot be incorporated into the abbreviated PDF article.

In our effort to encourage authors to write more balanced articles that fulfill the prescribed style, we ration the available space for each section. For instance, one third of the last page is strictly allocated to the Genetics and Expressions paragraphs. If an author chooses not to comment on those sections, that space will remain blank - to motivate authors to do something about it. Conversely, if the author provides more text than allowed, the surplus text will simply be trimmed to the nearest sentence.

The PDF feature was created to produce an article format that more closely resembles a 'journal paper', with pleasant typesetting and pagination. Indeed, many open source journals that publish exclusively online still invest significant resources to generate definitive PDF copies for all of their articles, even when HTML versions are adequate for practical purposes. We envision that for some users, once a TF has significantly piqued their interest for further perusal, they would be inclined to review the web version to access the most complete and up-to-date information.

Behind the scenes, our PDF rendering engine is based on in-house code and the dompdf 0.5.1 open source module. It uses fuzzy logic to handle the modifications necessary to determine the best solution of text, images, captions, and data tables to make the page layouts as aesthetically pleasing as 'machinely' possible. These modifications include changing the sizes of the figures, truncating excess text, reformatting the references, and calculating trade-offs between having larger figures and more data in data tables at the expense of less text, or keeping more text at the expense of having fewer figures and sparser data tables.

### Data export

One of the goals of TFe is to make TF data easily accessible to all. To support this goal, we built a web-based application programming interface (API) to facilitate a straightforward approach for extracting data from the TFe website. In addition to the TFe API, we have built a spreadsheet generator that allows visitors to download Excel (.xls) formatted files containing all of the information that is available through the web API, as a service for users who are not inclined to use the programmer-oriented web API. In short, virtually all forms of data available in TFe, including binding sequences, genomic targets, interactors, key papers, and even ontology terms, can be downloaded through the API, the spreadsheet generator, or PDF renderer. The TFe web-based API and its accompanying documentation can be found on the TFe website.

**Figure 9:**
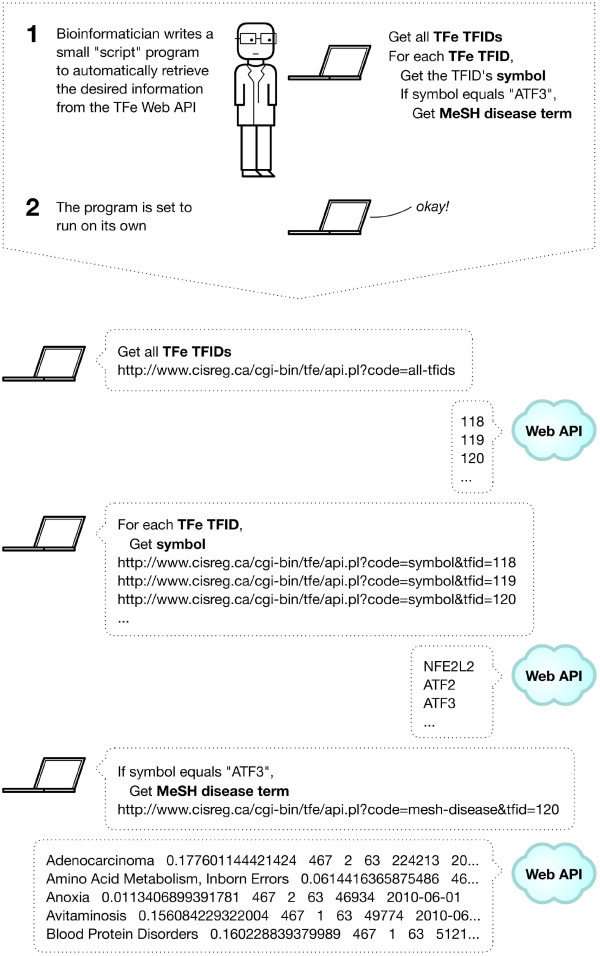
**Using the TFe web API**. Adventures in bioinformatics often involve large amounts of data retrieval and computation not amenable to manual labor. Thus, in place of humans, software is written to automate the grunt work, which may include computing vast quantities of data or obtaining large amounts of information from resources in the cloud, such as NCBI. To give researchers the option to retrieve data from TFe in an automated fashion, we have implemented a simple yet powerful web API. This figure provides a summary of what a data transaction may look like when using the TFe web API. In this case, the goal of the data retrieval exercise is to obtain all MeSH disease terms associated with the transcription factor 'ATF3'.

The presence of a machine-friendly API is what sets TFe apart from most other biomedical wikis. For easy parsing, the API sends data in tab-delimited plain text format. Since the API is web-based and communicates through the ubiquitous HTTP protocol, it is compatible with all common scripting and programming languages, including PHP Hypertext Preprocessor (PHP), Perl, and Python. See Figure 9 for an illustration of how the data retrieval process works when using the TFe web API.

### Article completion score

An article completion score (ACS) is automatically computed for every article in TFe. The ACS can range from 0% to 100%. Its purpose is to reflect the depth of annotation present in the article - the article's level of completeness. To illustrate, nearly complete articles typically have an ACS of 90% or more. The ACS is prominently displayed in the header of all articles on the TFe website in the form of a 'progress bar' that changes from orange to green as the score approaches 100% (Figure 5). The ACS system is designed to help authors determine whether their articles are sufficiently complete, and more importantly, identify article sections that are in need of more attention. By clicking on the 'see what's missing' link to the right of the progress bar, authors can view a list of suggestions that they can undertake to increase the score of their article, such as 'please provide more information in the Overview section of the Summary tab.'

The ACS evaluates the completeness of TFe articles based on several factors, which include, among others, the amount of text, figures, references, and data contributed for each article. Overall, 19 attributes (Table 3) are taken into account in the computation of the ACS.

**Table 3:** Computing the TFe Article Completion Score

Tab	Scoring element	Target	Points	Weight
Summary	Overview text	500 words	10 points	8.333%
Summary	References in overview text	3 references	5 points	4.167%
Summary	Figures	1 figure	10 points	8.333%
Structure	Overview text	200 words	5 points	4.167%
TFBS	Overview text	150 words	5 points	4.167%
TFBS	Binding site profiles	1 binding site profile	10 points	8.333%
Targets	Overview text	200 words	5 points	4.167%
Targets	Targets	10 targets in total (both author and auto)	10 points	8.333%
Protein	Isoforms text	200 words	5 points	4.167%
Protein	Covalent modifications text	200 words	5 points	4.167%
Interactions	Overview text	200 words	5 points	4.167%
Interactions	Ligands	1 ligand	1 point	0.833%
Interactions	Interactions	10 interactors in total (both author and auto)	10 points	8.333%
Interactions	Interactions	All 'nature of interaction' fields annotated	10 points	8.333%
Genetics	Overview text	250 words	5 points	4.167%
Expression	Overview text	200 words	5 points	4.167%
Papers	Papers	15 papers	10 points	8.333%
Papers	Papers	3 papers marked as 'recommended'	3 points	2.500%
(all)	Links	1 link	1 point	0.833%
			**120 points**	**100%**

The TFe Article Completion Score (ACS) is based on the 19 components listed here. Authors earn points for completing each component, up to the prescribed maximum for that component ('Maximum points'). So, for example, an author would be granted one point for adding one link, but no additional points would be granted if the author adds a second or third link. This prevents the author from adding 24 links to boost their ACS score by 20%. A 'fully complete' article would net 120 points, which gives a score of 100%.

**Figure 10:**
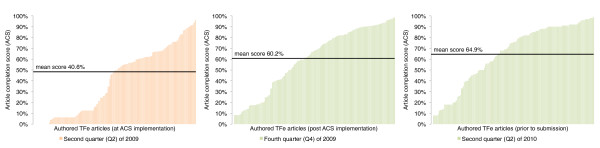
**The completion scores of authored articles in TFe**. The y-axis of this graph is the article completion score (ACS), while the bars on the x-axis represent the 176 authored TF articles in TFe (some of which are still works-in-progress), ordered such that higher scoring articles are positioned on the right (for clarity). In this graph, the completion scores of the 176 articles from three different periods - Q2 2009, Q4 2009, and Q2 2010 - are superimposed to demonstrate that the scores have been increasing over time. Within six months of the implementation of the ACS system in Q2 2009, the completion scores of authored TFe articles have increased from 40.6% to 60.2%, thus attesting to the effectiveness of this feedback mechanism (see Q2 2009 versus Q4 2009).

The ACS was implemented during TFe's beta testing period, when we observed that authors need guidance as to the expected level of article content. Prior to the implementation of the ACS, a large majority of 'completed articles' were deemed substantially incomplete. The ACS metric has established a standard for author contribution and helps authors attain this standard by highlighting sections of deficient articles that require further attention, and notifies authors how the deficiency can be remedied. While developed *de novo*, subsequent feedback indicates that the progress tracking scores are reminiscent of content tracking scores utilized in the LinkedIn social networking system. The response to the ACS has been positive. Within six months of implementation, the completion scores of all articles increased from 40.6% to 60.2% (Figure 10). The scoring metrics for computing the ACS are presented in Table 3.

### User authentication

The user authentication system of TFe, which handles the 'sign in' and 'sign out' functions, is built upon the Perl CGI::Session module. All account passwords stored in the TFe database are encrypted to safeguard the privacy and security of TFe users.

## Software

In this section, we discuss in technical detail the software that runs the TFe website, mainly its user interface and system architecture.

### Overview

The TFe software is a database-driven website application that runs the TFe website. For end users, the TFe website is an information resource where researchers can read peer-reviewed, expert-written summaries on pertinent TFs as well as obtain a wide variety of TF-related data, including binding sequences, genomic targets, and TF-to-disease associations. See Figure 6 for a complete list of all types of information available on TFe.

As previously discussed, the TFe website also features a password-protected user interface that allows expert authors to create and edit TF articles, upload data, report technical problems (that is, bugs), and submit anonymous peer reviews of other articles. It also features a built-in Customer Relationship Management (CRM)-like tool to help the administrators recruit new authors, as well as manage and communicate with the rest of the consortium. In short, the TFe website is a specialized and integrated software platform that has been custom-built to facilitate a community-curated TF wiki project.

### User interface

The TFe website, which can be accessed at [18], features a familiar and streamlined graphical user interface that is written in Extensible Hypertext Markup Language (XHTML) 1.0 Transitional, Cascading Style Sheets (CSS), and JavaScript.

On the homepage, a large 'universal' search box dominates the center of the screen (Figure 4a). This search box allows users to quickly access TFe's built-in search engine, which accepts 18 different types of queries, including gene symbols, fragments of binding sequences, and the names of researchers who are associated with particular TFs through their publication records. Alternatively, visitors can click on the 'go to a random article' link to view a random article on the article page.

Displayed in Figure 4b and as previously discussed, the article page is the centerpiece of the TFe-user interaction as it is where the bulk of TFe content lies. It features a compact yet informative and graphically rich header with key pieces of information about the TF, followed by the described ACS indicating the TF article's level of completeness or 'depth'. Below the ACS, the contents of the article are divided into ten tabs labeled 'Summary', 'Structure', 'TFBS', 'Targets', 'Protein', 'Interactions', 'Genetics', 'Expression', 'Ontologies', and 'Papers'. A row of navigation links is placed unobtrusively on the left side of the page. Other noteworthy pages on the TFe website include the classification page (Figure 4c) and the browse page. The classification page presents an organized hierarchy of TFs based on the TFCat [4] and the extended TF classification system of Vaquerizas *et al*. [5]. The browse page allows users to browse for TF articles based on various attributes such as name, classification, and level of completeness.

### System architecture

The TFe website software is written almost entirely in the Perl programming language, using the 'LAMP' (Linux, Apache, MySQL, Perl/PHP) paradigm for developing web-based applications. The Perl programming language was chosen for its robust text-manipulation capabilities and widespread support within the bioinformatics research community. In developing the website software, we have incorporated Perl and PHP modules and software packages to handle specialized tasks - such as reading the database, generating PDF files, and resizing images. See Table 4 for a list of Perl and PHP modules and software packages incorporated into the TFe software.

**Table 4:** Perl and PHP modules used in TFe

Language	Module	Purpose
Perl	CGI	Web browser interface
Perl	CGI::Session	User login
Perl	Crypt::Blowfish	Data encryption and random string generation
Perl	DBI	MySQL database interface
Perl	GD::Image	Creation of TF binding site diagrams
Perl	HTML::Detoxifier	User input filtering
Perl	Image::Resize	Image resizing and formatting
Perl	LWP::Simple	Interface between TFe and web-based APIs
Perl	pazar	Data retrieval from PAZAR
Perl	pazar::gene	Data retrieval from PAZAR
Perl	pazar::reg_seq	Data retrieval from PAZAR
Perl	TFBS::PatternGen::MEME	Creation of TF position weight matrices
PHP	dompdf 0.5.1	PDF generation

Listed on this table are the second and third party modules incorporated into TFe, with their respective programming languages, usage in TFe, and current web addresses at the time of publication.

The TFe website software is designed to run quickly and efficiently, yet remain relatively simple for programmers and system administrators to maintain. One challenge we had to overcome during the development process was keeping the software fast and responsive despite its size and complexity. One solution was to purposefully fragment the TFe website software into over 40 independent components. Each component serves a single unique purpose - for instance, to generate the home page, or to search the database, or to display articles. Each component can be summoned separately and without disturbing the other components. This fragmentation allows us to improve the speed and responsiveness of the TFe website, as at any given time only a fraction of the entire TFe software is being executed by the server.

**Figure 11:**
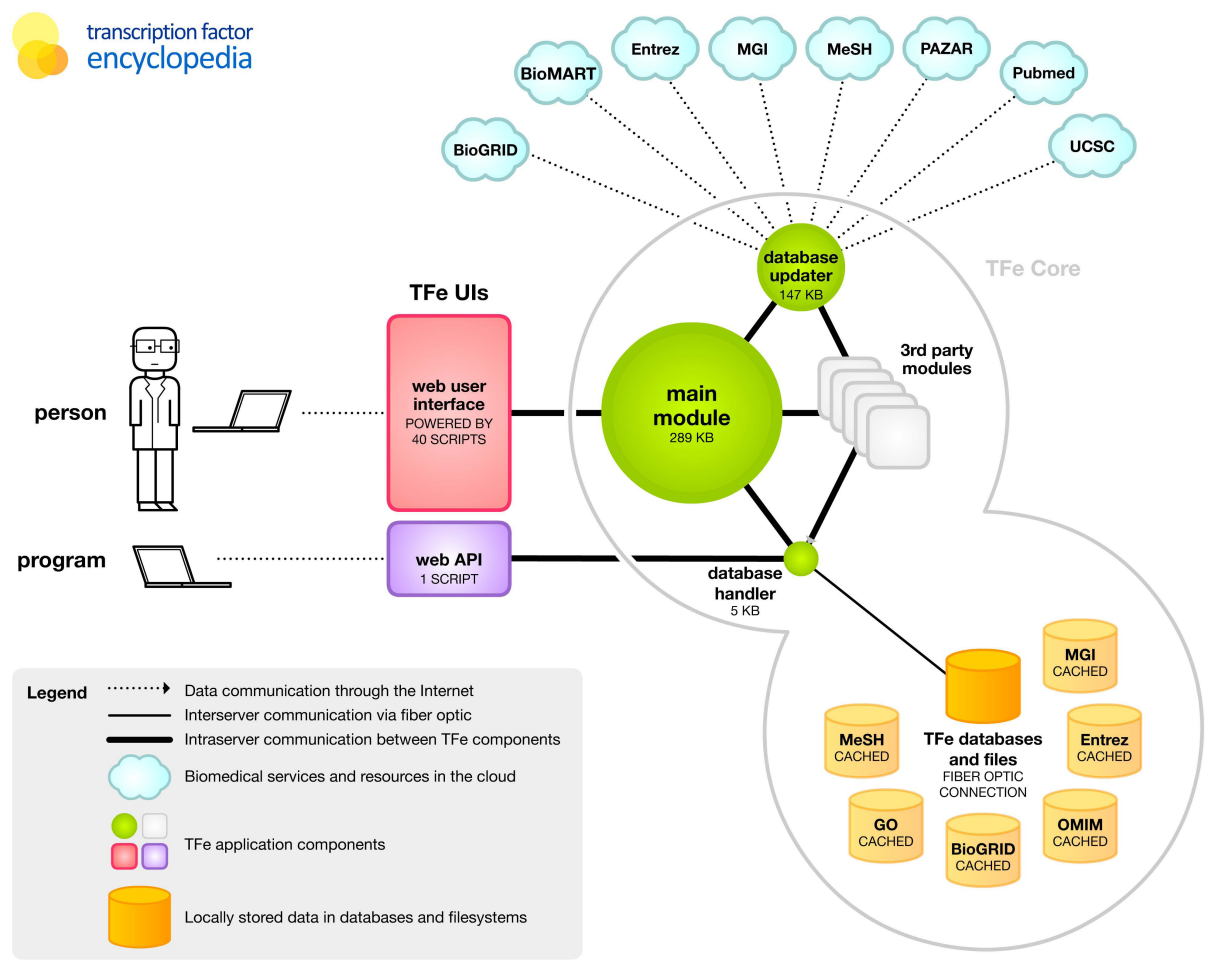
**Software architecture**. This schematic demonstrates the conceptual structure of the TFe software. Written mainly in the Perl programming language, the software is essentially a collection of Perl 'scripts' that runs on an Apache web server, in a UNIX-compatible environment. The software relies on MySQL for data storage, and a number of third party modules. Over 40 'front line' scripts (shown as the red rectangle) generate individual pages such as the home page and article page. These front line scripts are backed by a cluster of three TFe Perl modules (shown as the green circles): (1) the 'database updater', which is summoned *pro re nata *whenever the TFe database needs to be maintained or updated with new content from external sources such as NCBI; (2) the 'main module', which contains shared subroutines such as those that generate page headers; and (3) the 'database handler', which forms the gateway between all components of the TFe software and the TFe database. The database (shown as the yellow cylinder) is stored on a separate database server and communicates with the rest of the TFe software via fiber optic. It contains cached copies of third party resources so that the TFe software does not have to constantly retrieve data from the 'cloud'. This optimizes performance. The web API (shown as the purple rectangle) is directly connected to the ultra small and efficient database handler module. In bypassing activation of the large main module and database updater, the web API is able to run faster than the web-based interface. GO, Gene Ontology; MGI, Mouse Genome Informatics.

To reduce code repetition, we placed shared functions - such as those that generate the page header or navigation links - in a shared module that can be summoned by any component as needed. We call this module the 'TFe core module' because it forms the nucleus of the TFe website software. To further increase speed, we divided this TFe core module into three separate components: (1) a component that contains the vast majority of shared functions called 'tfe.pm'; (2) a component that contains only those functions involved with database reads and writes called 'db.pm'; and (3) a component that deals with maintenance and update functions called 'update.pm'. See Figure 11 for a schematic representation of the TFe website software.

With regards to hardware architecture, the TFe website software is currently implemented in Linux-based (CentOS) environment using a dedicated virtual server. The TFe software stores data in both the UNIX file system (that is, for images and PDF files) and a MySQL database, both of which are physically located in a proximal storage area network. To share computational load and optimize service responsiveness, a dedicated database server executes all complex database queries. This database server is connected to both the primary TFe web server and storage area network via fiber optic.

## Development process

The TFe system was developed over a period of three years. Early prototypes in 2007 were subjected to intense testing and continuous refinement by the programming team during what we refer to as the 'pre-alpha stage'. In the 2008 'alpha stage', external quality control testing was initiated by inviting ten authors to provide feedback on the software's design, features, and usability. By 2009, the software had evolved to a more stable and mature form. At this 'beta stage', we invited over 100 TF experts from around the world to contribute articles.

Over the next six months, TF experts responded to our invitations and began producing articles. To cope with the influx of feedback, we implemented an online feedback form. We upgraded our bug tracking process by adopting MantisBT, a web-based system that is available at [31]. All feedback was reviewed and prioritized for system modification if justified. Small changes were addressed immediately.

A rigorous backup regimen occurs on a daily and weekly basis to help us quickly and fully recover in the event of catastrophic system failure.

## Discussion

Three prominent systems have been introduced that rely more heavily on the community-contributed content wiki model. These are: (1) WikiProteins [32]; (2) WikiGenes [13]; and (3) Gene Wiki [14]. WikiProteins uses automated procedures to extract information from multiple resources, a text-based procedure to summarize these data, and a wiki-based format to collect user-supplied information. Similarly, WikiGenes uses a text-based procedure based on the iHOP service to present automated content organized under categorized subjects, and users are encouraged to provide content and corrections to the system, with their identities displayed to acknowledge contributions. Gene Wiki, the product of which resides within Wikipedia, automates the creation and maintenance of 'stub' articles on genes, thus creating a systematic framework for gene content. Despite the quality of these systems, examples of deep community commitment to contribute content are rare. By visual inspection, most entries in these systems still contain mostly automated content.

A striking divergence from the classic model is GeneTests [33], in which expert authors are recruited for each subject gene, taking intellectual ownership of an article of substantial importance to the clinical genetics community. When contrasting GeneTests to the aforementioned wiki-based systems, two qualities contribute prominently to the success of the former. First, GeneTests addresses a niche, allowing content to be tailored to the needs of a target audience. Second, the scientists who write articles on GeneTests are strongly acknowledged, allowing them to receive recognition for their intellectual contributions. While lasting participation in - and the continuing evolution of - GeneTests may ultimately derive from the intense commitment of the project's directors, it stands out as one of the rare cases in which prominent genetics researchers contribute original content to a community resource.

TFe represents a new direction in scientific communication of gene-specific information. Combining automated data presentation with expert-user reviews, the wiki-based system provides succinct reports about TFs, one of the most highly studied classes of proteins. The highly engaged efforts by researchers worldwide demonstrate that a wiki-based system can attract active participation and meet high quality standards of scholarly content. With over 100 mini reviews presented in the initial release, TFe represents one of the largest community participations in a gene-focused wiki project.

While the term wiki has become loosely applied over the years, in reality the term refers to a specific class of software that allows shared development of a document. However, in its most basic sense the term is commonly used to reflect the philosophy that information is best made accessible and editable by anyone *pro bono*. The wiki model has caught the attention of some scientists, who see it as a powerful tool that can hasten the pace of scientific communication. In the wake of Wikipedia's success, there emerged a high profile rallying call to create a gene-function wiki for scientists [34], and several groups have heeded this call by creating various scientific wikis, some built from the ground up [13] and some derived from existing general purpose wiki engines [32,35].

Unfortunately, as evidenced by WikiProteins [32], WikiGenes [13], and to a lesser extent Gene Wiki [14], scientific wikis have generally struggled to attract the level of community involvement envisioned by their founders. There are several contributing influences for the observed low rate of participation. The success of Wikipedia is in part attributable to the enthusiasm of a tiny fraction of the large global community of Internet users who are willing to contribute content. The scientific community with expertise on a specific topic, on the other hand, is small. Thus, even if the participation rate among these scientists remains comparable to the participation rate of the global community of Internet users who contribute content to Wikipedia, there would still be far fewer scientists contributing. To make matters worse for proponents of scientific wikis, scientists seem generally less willing to participate in these sorts of endeavors than the average user, reflecting perhaps the enormous demands on their time or the relative age of the experts. For many, their limited time is dedicated to rewarding tasks, such as performing experiments and reporting on the results in peer-reviewed journals. Earning new publications appears to be a strong motivator for many scientists. Few are willing to spend the same amount of time and effort to expand a wiki article that resides in the public domain and from which they would not receive any substantial credit.

Recognizing these constraints, a critical component of the success of TFe is the provision for authorship credit. Furthermore, we strive to actively identify and recruit authors, as opposed to waiting for contributors to contact us. Without addressing these two aspects, we doubt that we would be able to attain the same level of community involvement. Ultimately, the support of a journal willing to publish the resulting mini reviews in the form of this article (subject to passing a peer-review process) was a key motivator for many authors to participate in the project.

The retention of peer-review within the wiki-based article development process is scientifically critical. Readers of the system must hold high confidence in the quality of the reports. To meet this standard, all participating authors were encouraged to provide anonymous peer review reports for a set of articles. Approximately 40% of TFe authors participated in this voluntary peer review program as peer reviewers of other TFe articles.

Author identification was a challenge. We initially sought participation from existing collaborators and subsequently from peer referrals. During this early part of the project, we were able to recruit a core team of about ten authors who also became our *de facto *'alpha testers', thus allowing us to incorporate user feedback during the application development process. These authors - and eventually other authors as well - had significant input into the TFe system throughout its formation.

Given the large number of characterized TFs, we ultimately needed a larger-scale approach. To this end, we identified researchers who frequently appeared as the senior author in publications that discuss a specific human or mouse TF (using an automated analysis of articles in PubMed). Overall, 251 authors were individually contacted via email. About 59% (149 authors) agreed to participate, in addition to 10 authors who were directly invited at the outset of the project, and 2 authors who expressed interest and joined without invitation. About 65% of the participants developed articles sufficiently for inclusion in this report.

Moving forward, TFe can be expanded, advancing the effort to the ultimate goal of a high-quality article for every human TF. For the future, we plan to adopt a more targeted approach by working with communities of authors who represent specific structural groups of TFs (for example, nuclear receptors) or TFs that function within a specific biological context (for example, diabetes). Such efforts can be partnered with sponsoring journals that agree to reward the community efforts with a citable publication.

## Citing the resource

To cite TFe as a concept or software tool, cite this paper. To cite specific mini review articles found on the TFe website, please use the following format when possible:

Author(s) last name followed by initials: **< TF symbol in bold and proper capitalization >**. In Yusuf D *et al.*: **The Transcription Factor Encyclopedia**. *Genome Biology *2012, **13**:R24.

Example:

Bolotin E, Schnabl JM, Sladek FM: **HNF4a**. In Yusuf D *et al*.: **The Transcription Factor Encyclopedia**. *Genome Biology *2012, **13**:R24.

## Conclusions

TFe is a new web-based platform for facilitating the collection, evaluation, and dissemination of TF data. It is organized and curated by a consortium of TF experts from around the world whose goal is to develop concise mini review articles on pertinent human and mouse TFs. TFe contains a wealth of TF information consisting of both automatically populated and manually curated content. Over 100 released articles are currently available, with more to come. By offering multiple data export options that include the web API, the PDF generator, and spreadsheet generator, TFe strives to be a convenient and accessible resource. The TFe is available at http://www.cisreg.ca/tfe.

## Materials and methods

TFe is an amalgamation of several different and highly involved projects. For the sake of brevity, here we present only the most important key points regarding the materials and methods we employed in creating TFe. Thus, we selectively describe the materials and methods used in creating: (1) our TF classification system; (2) our TF binding profiles; (3) our TF protein structure predictions; and (4) our TF-to-disease associations. We describe the latter two in greater detail.

### Transcription factor classification system

With few exceptions, all TF genes and classification information in TFe were sourced from TFCat, a large collection of predicted and confirmed mouse TF genes [4]. This collection is based on Entrez Gene identifiers. However, not all TF genes described in TFCat were added to TFe, as TFe is focused on those TFs that bind directly to DNA in a sequence-specific fashion. Thus, with few exceptions, only TFs tagged with the function-based taxonomy of 'DNA-Binding: sequence specific' in TFCat were added. Ultimately, out of about 1,764 mouse TF genes catalogued in TFCat, 585 were suitable enough to be imported from TFCat to TFe.

TFs in TFe are organized into 'groups' and 'families' based on their DNA binding evidence and transcriptional activation functions. This method of TF classification is inherited from TFCat. 'Groups' of TFs represent the highest level of organization in this classification system. Within each group exist different 'families' of TFs. For nuclear receptors, this classification system is further extended with a 'subfamily' category. Placement of nuclear receptors within the subfamily category is guided by recommendations from the Nuclear Receptors Nomenclature Committee [36]. For a comprehensive list of the groups, families, and subfamilies that are represented in TFe, refer to Additional file 3.

### Transcription factor binding profiles

Most of the profiles in TFe are generated through manual curation. Binding site data from our authors are submitted via a web-based form. Submissions were processed by the curatorial staff of the PAZAR database who confirm the quality of the submitted information and enter the data into the TFe division of the PAZAR database. Authors may submit either genomic coordinates or TF binding motifs, such as those generated in selection and amplification experiments.

### Protein structure predictions

In summary, DNA binding transcription factors have been extensively studied and can be grouped according to a structural classification system [4]. For each of the small set of structural domains known to facilitate sequence-specific protein-DNA interactions, solved protein structures have been reported. Thus, it is feasible to produce homology-based models for many DNA-binding domains of proteins represented in TFe by using these solved protein structures as templates.

We generated a set of 202 predicted protein structures--homology-based predictions of the DNA binding domains of TFs. To do this, we developed a custom pipeline, written in Python, that incorporates two tools well-known in the realm of protein studies: HMMER [37] and Modeller [38]. Our protocol is based on the work of Morozov and Siggia [39], in which templates are selected to optimize similarity of DNA-binding residues. This method has been shown to increase modeling accuracy at the DNA-binding interface.

There are three main steps in generating the structural predictions: (1) building the template library; (2) finding a suitable template for each unsolved structure we would like to model; and (3) creating the structural prediction using the template as a guide.

#### Building the template library

We downloaded the entire RCSB PDB database [40] and the Protein Families (Pfam) Pfam-A HMMs database [41]. Using a custom Python script, we identified and extracted records from the PDB database that appear to contain a DNA binding domain and depict a protein-DNA binding interface (see Additional file 6 for a list of PDB records extracted). Each record is fragmented into one or more files, such that each file contains only one chain and the DNA residue. Using HMMER and the Pfam-A HMMs database, we analyzed each fragmented PDB record to catalogue all Pfam domains contained in the protein sequence. The result of this exercise is a list of relationships between Pfam domains and PDB records (Additional file 7). This constitutes our template library.

#### Finding a suitable template for each unsolved structure

For each unsolved TF protein structure, we looked for Pfam domains in the protein sequence by reviewing protein domain annotations provided by Entrez Gene. Since we are focused on modeling just the DNA binding domain of the TF protein, we removed the rest of the protein sequence. We then looked for templates in our PDB set that contain the same Pfam binding domains. We take these matching templates and compare each individually with our unsolved protein structure until the most suitable template is found. Our comparisons, which are done by an alignment tool, are scored based on similarity of the DNA-binding domain residues. For TFs known to form homodimers, a homodimeric template is selected.

#### Creating the structural prediction

After the most appropriate template is found, we input the unsolved protein sequence and the chosen template to Modeller 9v2, which constructs the predicted structure. After the structure is complete, we transfer the DNA residue from the template to the model by superimposing the two protein structures in three-dimensional space to find the most optimal superimposition, copying the DNA residue from the template to the model, and transposing the DNA residue per the superimposition coordinates. As mentioned earlier, the DNA molecules are stylistic and do not represent particular sequences - for example, the consensus sequence for the TF. The final predicted structure is rendered using iMol for presentation on the website.

### Transcription factor to disease associations

TFs are a class of proteins that are highly implicated in disease. Thus, we have made disease annotations an important component of TFe. Under the 'Genetics' section of all TFe articles, we have implemented a 'cloud' report of associated MeSH disease terms, along with their respective *P*-values. These annotations were generated in-house using a novel pipeline. Conceptually, the pipeline works as follows.

In PubMed, most - if not all - articles are tagged with a list of MeSH terms by NCBI curators. Some of these terms refer to diseases such as 'Diabetes Mellitus, Type 2' or 'Aniridia'. In addition, articles are often tagged with the identifiers of genes that are featured prominently in the report. Ultimately, an article is tagged with a list of MeSH terms, and also a list of genes. In TFe, we have leveraged these annotations to infer associations between TFs and certain diseases. For instance, mutations in the TF PAX6 are causal for the genetic disorder aniridia [42]. An automated analysis of all PAX6-referring articles identifies the term 'Aniridia' as appearing far more often than expected by chance (Fisher's exact *P*-value 3.2 × 10^-184^).

In the end, we generated 58,807 predicted TF-to-disease associations for the TFs in TFe (mean of 74.2 associations per TF) with a scoring threshold of 0.05. These associations can be viewed under the 'Genetics' tab on the TFe website. An overview of our approach is as follows.

#### Creating the associations

We derived these associations utilizing data from PubMed and Entrez Gene. In the PubMed database, the publications indexed in PubMed are associated with MeSH terms. For instance, in PubMed, a publication about the well-characterized gene *TP53 *may be associated with the MeSH terms 'Cell Line, Tumor', 'Oncogene Proteins, Fusion', 'Tumor Suppressor Protein p53', and the like. We refer to this set of data as 'mesh2pubmed' as it links MeSH terms to PubMed references.

In the Entrez Gene database, there similarly exists the 'gene2pubmed' dataset that associates PubMed references with genes. Given these resources, it is possible to create a link between genes and MeSH terms through PubMed references. The end result is a set of 'many-to-many' associations between MeSH terms and genes, such that each MeSH term is associated with numerous genes, and *vice versa*. As MeSH is a hierarchical controlled vocabulary maintained by curators and only the most specific relevant terms are ultimately associated with each PubMed article, each MeSH term is expanded to include all of its more generic parent terms. For instance, the MeSH term 'Diabetes Mellitus, Type 2' would be expanded to include 'Diabetes Mellitus', 'Glucose Metabolism Disorders', 'Metabolic Diseases', and 'Nutritional and Metabolic Diseases' (the latter being the broadest and most generic term).

Following this exercise we are left with millions of gene to MeSH associations, and - in particular - 662,163 associations between TF-encoding genes and MeSH terms. Yet, not all associations are informative. For instance, the MeSH term 'Humans' is associated with many genes and - in practice - offers little annotation value. On the other hand, the association - or multiple associations - of a relatively rare term such as 'Leukemia, Erythroblastic, Acute' with a TF-encoding gene may offer greater insight into the function of that gene.

To evaluate the quality of these associations, we computed Fisher's exact test *P*-value scores (Equation 1) for each TF to MeSH term association. In this equation, *n *is the number of articles associated with the gene via 'gene2pubmed'; *k *is the number of *n *articles associated with the gene annotated with the MeSH term; *N *is the number of articles in PubMed; and *m *is the number of articles in PubMed annotated with the MeSH term. For the background set, we compute the average rate of occurrence for all possible gene-to-MeSH term associations, taking into account not only whether an association exists, but also how often the same association occurs in each gene. In short, relatively rare MeSH terms that are associated multiple times with the same gene will yield a low (significant) *P*-value, while relatively common MeSH terms that are associated a few times with the same gene will yield a high (insignificant) *P*-value. We have not applied corrections for multiple hypothesis testing in our *P*-values, although we plan to implement this option in the future.

(1) $$\begin{array}{c} \text{Pr}\left(K\leq k\right)=\overset{k}{\underset{i=0}{\sum }}\frac{\left(\frac{m}{i}\right)\left(\frac{N-m}{n-i}\right)}{\left(\frac{N}{n}\right)} \end{array}$$

On the TFe website, these data are displayed to the user through the use of MeSH term clouds, where clicking on a term in the cloud launches a PubMed search displaying the relevant articles.

This procedure resulted in 662,163 TF to MeSH term associations, 333,909 of which return a *P*-value of ≤ 0.05. About 58,807 of these associations are of TFs in the TFe database. Overall, TFs are significantly linked to 2,121 out of over 4,400 disease terms described in the MeSH vocabulary.

## Abbreviations

ACS: article completion score; API: application programming interface; CIHR: Canadian Institutes of Health Research; MeSH: Medical Subject Headings; MSFHR: Michael Smith Foundation for Health Research; NCBI: National Center for Biotechnology Information; OMIM: Online Mendelian Inheritance in Man; PDB: Protein Data Bank; PDF: Portable Document Format; PHP: PHP Hypertext Preprocessor; RSCB: Research Collaboratory for Structural Bioinformatics; TF: transcription factor; TFBS: transcription factor binding site; TFCat: Transcription Factor Catalog; TFe: Transcription Factor Encyclopedia.

## Competing interests

The authors declare that they have no competing interests.

## Authors' contributions

WWW conceptualized and led the project. WWW, DY and SLB defined TFe content. DY designed, developed, and maintained the database, interface and software. WWW, SLB, MS and DY invited authors, corresponded with collaborators, and managed day-to-day operations. WWW, MS, WAC, SLB and DY devised and implemented the author recruitment and relationship management system. AT and PB envisioned and designed the structural prediction pipeline. AT, PB, and DY produced all native structural predictions. XYCZ and CTDD annotated the binding site profiles with the assistance of EP. WAC performed the TF to MeSH term association analysis. EP and SLB managed the process of importing binding site data into PAZAR for eventual use in TFe. DLF provided early suggestions on the creation of a wiki-based system and provided the TF classification system. DY and WWW drafted the manuscript with the assistance of WAC, AT, and SLB. All other authors contributed content and provided feedback. All authors read and approved the final manuscript for publication.

## Supplementary Material

Additional file 1 **PDF of released article**. The PDF version of the human FOXL2 article. Other PDF versions of released TFe articles can be accessed on the TFe website. Click here for file

Additional file 2 **Data of released articles**. Additional data related to the released mini review articles to supplement the four-page PDF versions, arranged in alphabetical order by TF name. Click here for file

Additional file 3 **Classification of transcription factors in TFe**. There are 803 human, mouse, and rat articles in TFe, most of which are organized into groups, families and subfamilies of TFs. The classification scheme utilized in TFe is derived from the work of Fulton *et al*. [5] in TFCat. There are 8 large groups, which are further subclassified into 34 families. Several TFs, namely nuclear receptors, are even further subclassified into subfamilies. Click here for file

Additional file 4 **The TFe article structure**. Articles in TFe are organized into ten tabs labeled 'Summary', 'Structure', 'TFBS', 'Targets', 'Protein', 'Interactions', 'Genetics', 'Expression', 'Ontologies', and 'Papers'. Each tab, with the exception of the Ontologies and Papers tabs, typically begins with a brief overview written by the authors, followed by a mixture of tables and figures that features data from both the authors and second (that is, PAZAR) or third party (that is, BioGRID) sources. Click here for file

Additional file 5 **Binding models produced in the TFe project**. Images of the binding models produced in TFe that are sufficiently characterized to be used in a study. Click here for file

Additional file 6 **PDB records depicting protein-DNA binding interface**. A list of PDB records that depict a protein-DNA binding interface. Click here for file

Additional file 7 **Relationship Between Pfam domains and PDB records**. A list of Pfam binding domains followed by PDB records in which the domains can be found. Click here for file
