# Supplementary material for: The Transcription Factor Encyclopedia
Source: Genome Biol. 2012 Mar 29;13(3):R24. doi: 10.1186/gb-2012-13-3-r24 (PMC3439975; doi:10.1186/gb-2012-13-3-r24)
Supplement: Additional file 1 — PDF of released article. The PDF version of the human FOXL2 article. Other PDF versions of released TFe articles can be accessed on the TFe website. [file gb-2012-13-3-r24-S1.PDF]

Updated April 30th, 2010
http://www.cisreg.ca/cgi-bin/tfe/articles.pl?tfid=436

FOXL2

Homo sapiens forkhead box L2

By Bérénice A. Benayoun[1] & Reiner A. Veitia[1*]

FOXL2 is a member of the superfamily of Forkhead box transcription factors, whose mutations are responsible for the Blepharophimosis Ptosis Epicanthus-inversus Syndrome in humans[1]. This rare genetic disorder is characterized by mild craniofacial defects, which can be isolated (BPES type I) or in association with premature ovarian failure (BPES type II)[2]. No clear genotype-phenotype relationship has been found between mutations and BPES type *a priori*, but a recent study suggests that mutations leading to BPES type I or II behave differently in functional reporter assays[3]. The BPES phenotype is nicely explained by the defects observed in the two different *Foxl2* knock-out mice models, though the invalidation models present a mostly unexplained high perinatal lethality[4,5]. FOXL2 is one of the earliest markers of ovarian determination, and its expression is maintained in ovarian granulosa cells from ovarian determination on, throughout female fertile life in Vertebrates[6]. A recent transcriptomic study in a granulosa cell model has suggested the involvement of FOXL2 in the regulation of cholesterol homeostasis, steroid metabolism, apoptosis, reactive oxygen species detoxification and inflammation/ovulation processes[7]. FOXL2 involvement in the cellular response to oxidative stress has been confirmed and studied more in-depth[8]. All of these processes are not equally affected by FOXL2 naturally-occurring BPES-causing mutations[9,8]. Interestingly, FOXL2 is a highly post-translationnally modified protein, modified by at least phosphorylation, acetylation as well as SUMOylation, and its target gene specificity may be fine-tuned in response to various signals, including cellular stress and sirtuin activation, by the induction of differential post-translational modification isoforms[10,8]. Interestingly, the specific FOXL2 response element (FLRE) is slightly divergent from other Forkheads', which is compatible with its unique role in gonad primordium determination towards ovarian development[11]. Although FOXL2 expression pattern has not been extensively characterized, FOXL2 has also been involved in the organogenesis and function of the pituitary, where it is expressed mainly in thyreotrope and gonadotrope cells. Its described targets in this organ are mainly involved in the regulation of gonatrophins secretion (transcriptional regulation of the GnRH receptor[12], of the alpha-Glycoprotein Hormone Subunit (alpha-GSU)[13], of the beta subunit of FSH[14] and of Follistatin[15]. To regulate *GnRHR* and *Follistatin* expression, FOXL2 has been shown to cooperate by direct binding with Smad3, a downstream effector transcription factor under the regulation of the TGF-beta cytostatic pathway[15,13]. Interestnigly, two recent studies have suggested a potential role for FOXL2 in the regulation of ovarian granulosa cell tumorigenesis: indeed, the first study found its expression was either lost or reduced in the most aggressive cases, and the second study identified a recurring somatic mutation in over 97% of the tumors[16,17]. *(continued on site)*

Binding sites

FOXL2 binds with high affinity the FOXL2 Response Element (FLRE), slightly divergent from the general binding consensus of Forkhead factors. Whereas the general consensus site is 5-(G/A)(T/C)(A/C)AA(C/T)A-3, a high-affinity binding site consensus for FOXL2 was recently identified as 5-GT(C/G)AAGG-3[11]. The FLRE has been shown to be enriched in the promoters of FOXL2 potential transcriptional targets[11]. FOXL2 also seems to be also able to bind elements diverging from the FLRE and closer in sequence to more conventional Forkhead binding consensus, albeit with a lesser affinity: indeed, a mutation of GG to TT greatly diminishes FOXL2 transactivation potency, without abolishing it[11]. Moreover, FOXL2 was shown to transactivate the promoter of the *GnRHR* gene using the sequence 5'-CACAACA-3', closer to the general consensus (no final GG)[12], and the promoter of the *FST* gene using the sequence 5-ATCAATGT-3[15], which presents similarities with both consensus. *(continued on site)*

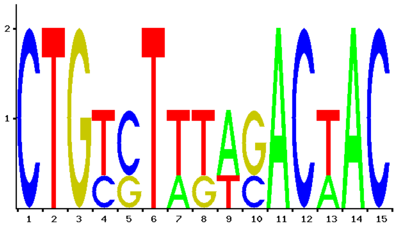

Binding profile from Pazar

Project name	TFe
TF name	FOXL2_MOUSE
TF species	*None*
Pazar ID	TF0000786
Ensembl ID	ENSMUST00000051312

This data is sourced from Pazar, a public database of transcription factor and regulatory sequence annotation. http://www.pazar.info/

PFM	1	2	3	4	5	6	7	8	9	10	11	12	13	14	15
A	0	0	0	0	0	0	1	0	2	0	3	0	1	3	0
C	3	0	0	1	2	0	0	0	0	1	0	3	0	0	3
G	0	0	3	0	1	0	0	1	0	2	0	0	0	0	0
T	0	3	0	2	0	3	2	2	1	0	0	0	2	0	0

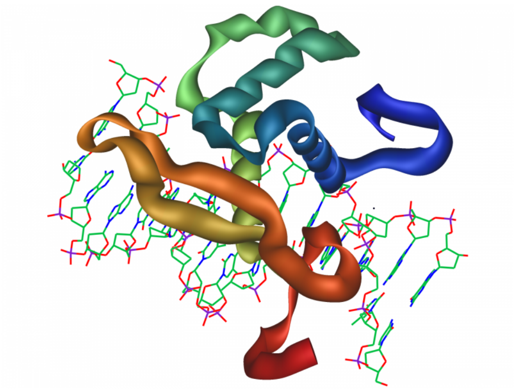

Protein structure of FOXL2

Although the particular 3D-structure of the Forkhead transcription factor FOXL2 has not been elucidated yet, sequence homology and bioinformatical models suggests the structure of its DNA-binding domain is highly similar to that of other Forkhead box transcription factors[1,18,11]. At the C-terminus of the FKH sequence, FOXL2 possesses two NLS sequences (one atypical and one typical RK-rich) that promote its constitutive nuclear localization[19]. FoxL2 proteins are more divergent outside of their DNA-binding domain, though a high degree of conservation is still observed, suggesting evolutionary constraints[6,20]. The molecular functions or structure, if any, of these protein regions is still widely unexplored. An easily recognizable domain of FOXL2 is a polyAlanine tract, whose length (14 repeats) is strictly conserved among eutherian mammals, but absent in birds and fish[20]. The role and structure of FOXL2 polyalanine domain is unknown, but expansions of this domain are pathogenic, and represent about 30% of FOXL2 mutations in BPES patients[21,9]. Polyalanine expansions of FOXL2 have been shown to induce cytoplasmic and intranuclear cellular aggregation of the protein, as well as perturbations of protein solubility in COS-7 cells[21,9].

Classification

Group	Winged Helix-Turn-Helix
Family	Forkhead Domain Family
Subfamily	Not specified

Resources

Entrez Gene	668
Ensembl	ENSG00000183770,
Refseq	NP_075555
Uniprot	P58012
OMIM	608996, 605597, 110100
Synonyms	POF3, PINTO, PFRK, BPES1, BPES

About

© 2012 Benayoun *et al*. This is an open access article distributed under the terms of the Creative Commons Atribution-Share Alike 3.0 Unported License, which permits unrestricted use, distribution, and reproduction in any medium, provided the original work is properly cited and that any work derived from it is distributed under the same, similar or a compatible license.

Contact

Affiliation(s): (1) Department of Molecular and Cellular Pathology, Institut Jacques Monod, Université Paris Diderot - Paris 7, Paris, Île-de-France, France. (*) Correspondence: Reiner A. Veitia. Email: reiner.veitia@inserm.fr

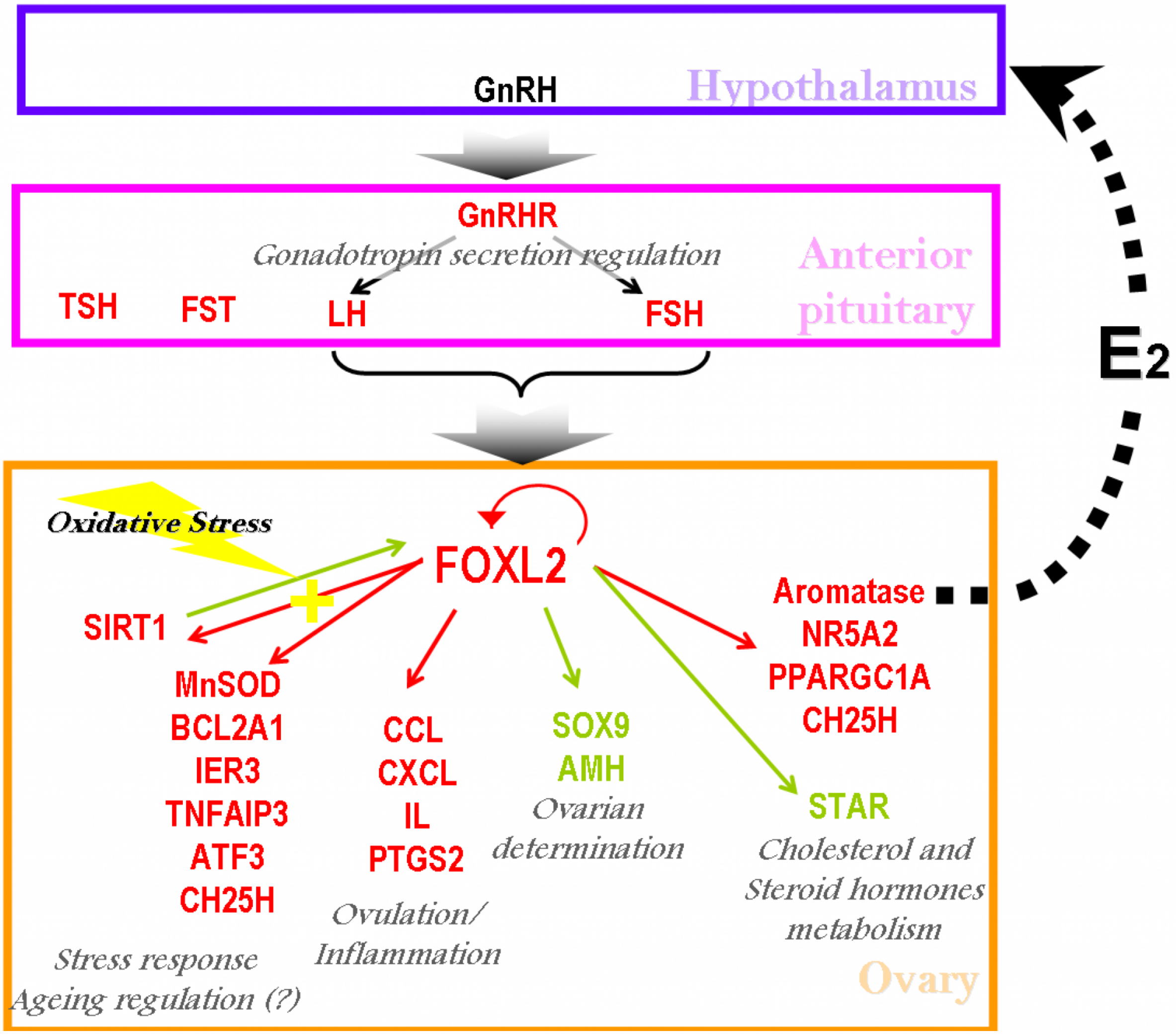

FIGURE 1 (1625) | **FOXL2, a master regulator of the hypothalamus-pituitary-ovarian axis in females.** Red text/arrows indicate genes activated by FOXL2 (including itselff). Green text/arrows indicates inhibition by FOXL2, directly or indiretly (the green arrow from SIRT1 to FOXL2 indicates the indirect negative feeback regulation that FOXL2 exerts on itself through activation of SIRT1). Black text indicates indirect regulation or no regulation. The three crucial compartment for reproduction in females are shown in boxes: the hypothalamus, which controls gonadotropin secretion through pulsatile production of GnRH, the anterior pituitary, which contains the FOXL2-expressing thyreotrope and gonadotropes cells and regulates folliculogenesis and ovulation through LH and FSH secretion, and the ovary, the female reproductive organ, which, in turn, regulates GnRH secretion by the hypothalamus via the production of Estrogens (E2) by the CYP19A1 aromatase enzyme (activation or inhibition according to the time of the menstrual cycle). Oxidative stress, which is figured here by the 'activating' yellow lightening, has been shown to enhance FOXL2 transactivation capacity on stress response genes in granulosa ovarian cells. This scheme recapitulates FOXL2 key position in the hypothalamus-pituitary-ovay axis. GnRH: Gonadotropin Releasing Hormone, GnRHR: GNRH Receptor, LH: Luteinizing Hormone, FSH: Folliculo-Stimulating Hormone, FST: follistatin, TSH: Thyroid Stimulating Hormone, MnSOD: mitochondrial Manganese Superoxide Dismutase, IL: interleukin, AMH: Anti-Müllerian Hormone, STAR: Steroidogenic Acute Regulatory gene, E2: estrogens.

Isoforms

Consistently with the fact that *FOXL2* is a monoexonic gene[1], only one mature protein isoform, post-translational modifications notwithstanding, has been described so far *in vivo*[6]. However, overexpression experiments in the heterologous COS-7 cells followed by Western Blot experiments have shown that FOXL2 mRNA could potentially harbour a IRES, leading to an initiation of translation at Methionine 137 (M137), whose relevance *in vivo* is yet to be determined[22].

Covalent modifications

FOXL2 has been shown to possess a rich pattern of post-translational modification (PTM) isoforms both in human granulosa-like KGN cells and in mice whole ovaries through 2D-Western Blot experiments[10]. Indeed, in KGN cells, at least 11 distinct PTM isoforms of FOXL2 coexist in the steady state. FOXL2 PTM isoforms are contained in two distinct trains of modification, a basic poorly modified train and a more acidic hypermodified train, separated by a pI (Isoeletric point) leap, with a remarkable absence of modification intermediates. Some modification pathways are mutually exclusive, suggesting that co-existing PTM isoforms are likely to be functionally non-equivalent[10]. FOXL2 has been shown to be modifiable by phosphorylation, acetylation and SUMOylation[10,8]. SIRT1 activation induces deacetylation and 'alkalinisation' of FOXL2 PTM isoforms, whereas oxidative stress favors hyperacetylation and reveals a SUMO1-conjugate isoform. The exact position of the residues actually modified in FOXL2 protein sequence have not yet been mapped but, interestingly, several BPES-causing FOXL2 mutations, and one described in an isolated POF case, alter potentially modifiable residues[23,24]. *(continued on site)*

Targets

In the context of the pituitary, FoxL2 has been shown to regulate the expression of genes involved in the production and the regulation of secretion of pituitary hormones. Indeed, FoxL2 regulates the transcription of the *alphaGSU* gene[13], which encodes the common subunit of all pituitary glycoprotein hormones, namely the Thyroid Stimulating Hormone TSH, and the gonadotropins LH (Luteinizing Hormone) and FSH (Follicle Stimulating Hormone). More recently, it was also shown that FoxL2 can activate the transcription of the *Fshb* gene, which encodes the beta subunit of FSH[14]. The secretion of gonadotropins by gonadotrope cells is triggered by the binding of the GnRH secreted by the hypothalamic neurons to its receptor on gonadotropes, and the secretion of LH and FSH is a function of both the amount of GnRH secreted and of the amount of GnRH receptor (GnRHR) expressed at the plasma membrane. FoxL2 is also able to regulate the secretion of gonadotropins at another level, through its transcriptional activation of the *GnRHR* gene[12]. In the ovary, FoxL2 seems to control ovarian differentiation through its transcriptional inhibition of *SOX9* and *AMH*, which are both male-promoting factors[25,26,7]. *(continued on site)*

Interactions

Foxl2 has been shown to be able to form heterodimers with the final TGFbeta pathway transducer Smad3[12,15]. The Foxl2-Smad3 complex was found to form a higher order complex with AP-1 on a complex regulatory DNA motif, the GnRHR Activating Sequence (GRAS), to promote transcription from the *GnRHR* gene[12]. The Tilapia ortholog of FoxL2 was proven to interact with Ad4BP/SF-1 (NR5A1), thus forming a functional heterodimer, which promotes *Cyp19a1* aromatase transcription[29]. Although the functional relevance of the finding is not clear yet, FOXL2 was recently shown to be able to form homodimers in an heterologous cell system (CHO cells)[14]. A 2005 study that described the ability of FoxlL2 to promote apoptosis in heterologous CHO cells found that this ability was achieved through direct interaction with dead-box helicase protein DP103/DDX20, although the precise mechanistic details involved was not elucidated[31]. FOXL2 has been found to be a SUMO1 conjugation substrate[8]. Finally, direct interaction with deacetylase SIRT1 has been suggested because the consequence of the overexpression of SIRT1 in cells naturally expressing FOXL2 is the deacetylation of endogenous FOXL2[10]. *(continued on site)*

TABLE 1. Key genomic targets and regulators of FOXL2

Displaying the first 24 of 45 records. See more on site »

Type	Gene	Gene ID	TF complex	Reference	Source
Target	Human **AMH**	EG 268	(not provided)	17728319	**Author** (all)
Target	Human **ATF3**	EG 467	(not provided)	17360647	**Author** (all)
Target	Human **ATF3**	EG 467	(not provided)	19010791	**Author** (BAB)
Target	Mouse **Amh** 00	EG 11705	(not provided)	15731305	**Author** (all)
Target	Mouse **Amh** 00	EG 11705	(not provided)	15944199	**Author** (BAB)
Target	Human **BCL2A1**	EG 597	(not provided)	17360647	**Author** (all)
Target	Human **BCL2A1**	EG 597	(not provided)	19010791	**Author** (BAB)
Target	Human **CCL20**	EG 6364	(not provided)	17360647	**Author** (all)
Target	Human **CCL3L1**	EG 6349	(not provided)	17360647	**Author** (all)
Target	Human **CCL3L3**	EG 414062	(not provided)	17360647	**Author** (all)
Target	Human **CCL3**	EG 6348	(not provided)	17360647	**Author** (all)
Target	Human **CH25H**	EG 9023	(not provided)	17360647	**Author** (all)
Target	Human **CH25H**	EG 9023	(not provided)	19010791	**Author** (BAB)
Target	Human **CXCL2**	EG 2920	(not provided)	17360647	**Author** (all)
Target	Human **CXCL3**	EG 2921	(not provided)	17360647	**Author** (all)
Target	Human **CYP17A1**	EG 1586	FOXL2-SF1	20207836	**Author** (BAB)
Target	Human **CYP19A1**	EG 1588	(not provided)	16720712	**Author** (all)
Target	Mouse **Cga**	EG 12640	(not provided)	16840539	**Author** (all)
Target	Rat **Dmrt1**	EG 114498	(not provided)	19264703	**Author** (all)
Target	Human **FOS**	EG 2353	(not provided)	17360647	**Author** (all)
Target	Human **FOXL2**	EG 668	(not provided)	18158309	**Author** (BAB)
Target	Human **FOXL2**	EG 668	(not provided)	18635577	**Author** (all)
Target	Human **FSHB**	EG 2488	(not provided)	19324968	**Author** (all)
Target	Sheep **FSHB**	EG 443387	(not provided)	19324968	**Author** (all)

TABLE 2. Interactors of FOXL2

Interactor	Nature of interaction (from author)	Experimental validation	Reference	Source
Mouse **Ddx20**	Not specified	Two-hybrid	16153597	**Author** (BAB)
Human **FOXL2**	Unknown	Co-purification	19324968	**Author** (BAB)
Mouse **Jun**	Physical: with another TF: complex binds DNA	Two-hybrid	12943993	**Author** (BAB)
Human **LATS1**	Physical: enzyme modification: phosphorylation	Co-purification	20407010	**Author** (BAB)
Mouse **Lats1**	Unknown	Two-hybrid	(not provided)	**Author** (BAB)
Human **NR5A1**	Physical: with another TF	Two-hybrid	17192407	**Author** (BAB)
Human **PIAS1**	Physical: enzyme modification: sumoylation	Co-purification	20209145	**Author** (BAB)
Human **SIRT1**	Physical: deacetylation	Not specified	19010791	**Author** (BAB)
Human **SUMO1**	Physical: enzyme modification: sumoylation	Co-purification	19010791	**Author** (BAB)
Mouse **Smad3**	Physical: with another TF: complex binds DNA	Co-purification	19106105	**Author** (BAB)
Mouse **Smad3**	Physical: with another TF: complex binds DNA	Two-hybrid	12943993	**Author** (BAB)
Human **UBE2I**	Physical: enzyme modification: sumoylation	Co-purification	20209145	**Author** (BAB)

Genetics

FOXL2 mutations are responsible for the Blepharophimosis Ptosis Epicanthus-inversus Syndrome (BPES; MIM 110100)[1]. This genetic disorder (prevalence less than 1/5000 births) is characterized by eyelid malformations, including small palpebral fissures, epicanthus-inversus, eyelids ptosis and a flat nasal bridge. Malformations can be associated with premature ovarian failure (POF), defining 2 types of BPES: BPES type I (with POF), and BPES type II (isolated eyelid defects)[2]. BPES was long considered an autosomal dominant disease, but a case of recessive BPES in a large consanguineous Indian family has been described[32]. Numerous mutations of *FOXL2* leading to BPES have been described. Described intragenic *FOXL2* mutations include expansions of its polyalanine domain (30% of cases), missense mutations (mostly in the Forkhead domain), nonsense mutations, and insertions/deletions leading to premature stop or aberrant elongated proteins[33]. No clear genotype/phenotype correlation can be established to explain how mutations can lead to BPES type I or II. Results from functional studies of FOXL2 mutated variants reveal that protein aggregation is a major pathogenic mechanism and loss of function is often found on reporter systems in consequence to *FOXL2* mutations, in a promoter-dependent manner[9,23,18,11,8]. *(continued on site)*

Expression

FOXL2 expression has mainly been detected in the developing eyelids as well as in fetal and adult ovaries[1,6,36]. In developing eyelids, FOXL2 is expressed in the primordial mesenchyma, which is consistent with the atrophy of the eyelid superior levator muscle observed in BPES patients[37]. FOXL2 expression begins early in development during the period of ovarian determination in genital crests and is maintained throughout adulthood in mammals. FOXL2 expression seems restricted to the somatic compartment, with a strong expression in granulosa cells. Foxl2 is also expressed ventrally in the developing pituitary, the Rathke's pouch, and probably participate in its organogenesis[13,38]. In the adult pituitary, its expression is found essentially in gonadotrope and thyreotrope cells [13]. Although the expression pattern of FoxL2 has not been extensively characterized outside of the craniofacial and gonadal regions, transcriptomic data suggests that its expression pattern may be wider than initially assumed. Indeed, an exploration of the GEO database suggests an expression at least at the RNA level in the heart (GDS2614), macrophages (GDS2686; GDS2041), circulating blood reticulocytes (GDS2655), colon (GDS756; GDS3226; GDS1780), hepatocytes (GDS1729; GDS2766; GDS2239), and bronchial muscle cells (GDS2628). FoxL2 expression at the protein level in these organs/cells would have to be confirmed, and its relevance remains to be explored.

Ontologies

Displaying 23 of 346 key TF-to-MeSH associations. Numbers indicate Fisher's exact test p-value. See more on site »

Blepharophimosis 2.4×10^{-130} Eye Abnormalities 1.1×10^{-86} Eyelid Diseases 1.0×10^{-83} Blepharoptosis 4.2×10^{-50} Congenital Abnormalities 1.8×10^{-44} Ovarian Failure, Premature 6.1×10^{-44} Eye Diseases 3.7×10^{-41} Syndrome 2.3×10^{-37} Disease 6.0×10^{-36} Congenital, Hereditary, and Neonatal Diseases and Abnormalities 1.6×10^{-33} Ovarian Diseases 1.4×10^{-26} Adnexal Diseases 1.5×10^{-25} Gonadal Disorders 1.7×10^{-24} Genital Diseases, Female 1.4×10^{-16} Endocrine System Diseases 5.8×10^{-13} Female Urogenital Diseases 5.6×10^{-11} Pathologic Processes 1.0×10^{-9} Female Urogenital Diseases and Pregnancy Complications 2.9×10^{-9} Pathological Conditions, Signs and Symptoms 5.0×10^{-6} Granulosa Cell Tumor 1.1×10^{-5} Abnormalities, Multiple 4.6×10^{-5} Sex Cord-Gonadal Stromal Tumors 9.9×10^{-5} Neoplasms, Gonadal Tissue 0.00010

References

1 Crisponi L *et al*. **The putative forkhead transcription factor FOXL2 is mutated in blepharophimosis/ptosis/epicanthus inversus syndrome.** *Nat Genet* 27(2).

2 Zlotogora J *et al*. **The blepharophimosis, ptosis, and epicanthus inversus syndrome: delineation of two types.** *Am J Hum Genet* 35(5).

3 Dipietromaria A *et al*. **Towards a functional classification of pathogenic FOXL2 mutations using transactivation reporter systems.** *Hum Mol Genet* 18(17).

4 Uda M *et al*. **Foxl2 disruption causes mouse ovarian failure by pervasive blockage of follicle development.** *Hum Mol Genet* 13(11).

5 Schmidt D *et al*. **The murine winged-helix transcription factor Foxl2 is required for granulosa cell differentiation and ovary maintenance.** *Development* 131(4).

6 Cocquet J *et al*. **Evolution and expression of FOXL2.** *J Med Genet* 39(12).

7 Batista F *et al*. **Potential targets of FOXL2, a transcription factor involved in craniofacial and follicular development, identified by transcriptomics.** *Proc Natl Acad Sci USA* 104(9).

8 Benayoun BA *et al*. **Positive and negative feedback regulates the transcription factor FOXL2 in response to cell stress: evidence for...** *Hum Mol Genet* 18(4).

9 Moumné L *et al*. **Differential aggregation and functional impairment induced by polyalanine expansions in FOXL2, a transcription factor involved in...** *Hum Mol Genet* 17(7).

10 Benayoun BA *et al*. **The post-translational modification profile of the forkhead transcription factor FOXL2 suggests the existence of parallel processive/concerted...** *Proteomics* 8(15).

11 Benayoun BA *et al*. **The identification and characterization of a FOXL2 response element provides insights into the pathogenesis of mutant...** *Hum Mol Genet* 17(20).

12 Ellsworth BS *et al*. **The gonadotropin releasing hormone (GnRH) receptor activating sequence (GRAS) is a composite regulatory element that interacts...** *Mol Cell Endocrinol* 206(1-2).

13 Ellsworth BS *et al*. **FOXL2 in the pituitary: molecular, genetic, and developmental analysis.** *Mol Endocrinol* 20(11).

14 Lamba P *et al*. **A novel role for the forkhead transcription factor FOXL2 in activin A-regulated follicle-stimulating hormone beta subunit...** *Mol Endocrinol* .

15 Blount AL *et al*. **FoxL2 and Smad3 Coordinately Regulate Follistatin Gene Transcription.** *J Biol Chem* 284(12).

16 Shah SP *et al*. **Mutation of FOXL2 in granulosa-cell tumors of the ovary.** *N Engl J Med* 360(26).

17 Kalfa N *et al*. **Extinction of FOXL2 expression in aggressive ovarian granulosa cell tumors in children.** *Fertil Steril* 87(4).

18 Nallathambi J *et al*. **Differential functional effects of novel mutations of the transcription factor FOXL2 in BPES patients.** *Hum Mutat* 29(8).

19 Moumné L *et al*. **The mutations and potential targets of the forkhead transcription factor FOXL2.** *Mol Cell Endocrinol* 282(1-2).

20 Cocquet J *et al*. **Structure, evolution and expression of the FOXL2 transcription unit.** *Cytogenet Genome Res* 101(3-4).

21 Caburet S *et al*. **A recurrent polyalanine expansion in the transcription factor FOXL2 induces extensive nuclear and cytoplasmic protein aggregation.** *J Med Genet* 41(12).

22 Moumné L *et al*. **Deletions in the polyAlanine-containing transcription factor FOXL2 lead to intranuclear aggregation.** *Hum Mol Genet* 14(23).

23 Beysen D *et al*. **Missense mutations in the forkhead domain of FOXL2 lead to subcellular mislocalization, protein aggregation and impaired...** *Hum Mol Genet* 17(13).

24 Harris SE *et al*. **Identification of novel mutations in FOXL2 associated with premature ovarian failure.** *Mol Hum Reprod* 8(8).

25 Ottolenghi C *et al*. **Foxl2 is required for commitment to ovary differentiation.** *Hum Mol Genet* 14(14).

26 Ottolenghi C *et al*. **Loss of Wnt4 and Foxl2 leads to female-to-male sex reversal extending to germ cells.** *Hum Mol Genet* 16(23).

27 Pisarska MD *et al*. **Forkhead l2 is expressed in the ovary and represses the promoter activity of the steroidogenic acute...** *Endocrinology* 145(7).

28 Pannetier M *et al*. **FOXL2 activates P450 aromatase gene transcription: towards a better characterization of the early steps of mammalian...** *J Mol Endocrinol* 36(3).

29 Wang DS *et al*. **Foxl2 up-regulates aromatase gene transcription in a female-specific manner by binding to the promoter as well...** *Mol Endocrinol* 21(3).

30 Oshima Y *et al*. **Molecular cloning and gene expression of Foxl2 in the frog Rana rugosa.** *Gen Comp Endocrinol* 159(2-3).

31 Lee K *et al*. **Transcriptional factor FOXL2 interacts with DP103 and induces apoptosis.** *Biochem Biophys Res Commun* 336(3).

32 Nallathambi J *et al*. **A novel polyalanine expansion in FOXL2: the first evidence for a recessive form of the blepharophimosis...** *Hum Genet* 121(1).

33 Beysen D *et al*. **FOXL2 mutations and genomic rearrangements in BPES.** *Hum Mutat* 30(2).

34 Gersak K *et al*. **A novel 30 bp deletion in the FOXL2 gene in a phenotypically normal woman with primary...** *Hum Reprod* 19(12).

35 Laissue P *et al*. **Functional evidence implicating FOXL2 in non syndromic premature ovarian failure and in the regulation of the...** *J Med Genet* .

36 Pailhoux E *et al*. **A 11.7-kb deletion triggers intersexuality and polledness in goats.** *Nat Genet* 29(4).

37 Dollfus H *et al*. **Sporadic and familial blepharophimosis - ptosis-epicanthus inversus syndrome: FOXL2 mutation screen and MRI study of the superior...** *Clin Genet* 63(2).

38 Treier M *et al*. **Multistep signaling requirements for pituitary organogenesis in vivo.** *Genes Dev* 12(11).

39 Lei N *et al*. **Distinct Transcriptional Mechanisms Direct Expression of the Rat Dmrt1 Promoter in Sertoli Cells and Germ Cells...** *Biol Reprod* .

40 Shi B *et al*. **Association of reproductive performance with SNPs of FOXL2 gene by SSCP in Japanese flounder (Paralichthys olivaceus).** *Comp Biochem Physiol B* Biochem. Mol. Biol..

41 Beysen D *et al*. **Identification of 34 novel and 56 known FOXL2 mutations in patients with Blepharophimosis syndrome.** *Hum Mutat* 29(11).

42 Baron D *et al*. **Foxl2 gene and the development of the ovary: a story about goat, mouse, fish and woman.** *Reprod Nutr Dev* 45(3).

43 Beysen D *et al*. **Deletions involving long-range conserved nongenic sequences upstream and downstream of FOXL2 as a novel disease-causing mechanism...** *Am J Hum Genet* 77(2).

44 Pannetier M *et al*. **Ovarian-specific expression of a new gene regulated by the goat PIS region and transcribed by a...** *Genomics* 85(6).
