# Supplementary material for: The Transcription Factor Encyclopedia
Source: Genome Biol. 2012 Mar 29;13(3):R24. doi: 10.1186/gb-2012-13-3-r24 (PMC3439975; doi:10.1186/gb-2012-13-3-r24)
Supplement: Additional file 4 — The TFe article structure. Articles in TFe are organized into ten tabs labeled 'Summary', 'Structure', 'TFBS', 'Targets', 'Protein', 'Interactions', 'Genetics', 'Expression', 'Ontologies', and 'Papers'. Each tab, with the exception of the Ontologies and Papers tabs, typically begins with a brief overview written by the authors, followed by a mixture of tables and figures that features data from both the authors and second (that is, PAZAR) or third party (that is, BioGRID) sources. [file gb-2012-13-3-r24-S4.PDF]

Tab	Sections	Description	Sources
Summary	Overview	A 500-word introduction	Authors
	Figures	One or more figures to describe key roles	Authors
Structure	Overview	A 200-word paragraph on protein structure	Authors
	Structures	Three-dimensional models of structural predictions	TFe, with templates from the RCSB Protein Data Bank
	Family	A list of other transcription factors occupying the same family classification	TFCat
	Figures	One or more figures to describe important aspects of protein structure	Authors
TFBS	TFBS logos	A collage of binding site profile thumbnails	PAZAR
	Overview	A 200-word paragraph on binding sites and binding activity	Authors
	Binding site profiles	A list of binding site logos, position frequency matrices (PFMs), and sequences	PAZAR
Targets	Overview	A 200-word paragraph on genomic targets	Authors
	Targets (author curated)	A list of genomic targets with Pubmed references and associated biological process GO terms	Authors
	Targets (automatically populated)	A list of genomic targets with Pubmed references and associated biological process GO terms	PAZAR
Protein	Isoforms	A 200-word paragraph on isoforms	Authors
	Covalent modifications	A 200-word paragraph on covalent modifications	Authors
Interactions	Overview	A 200-word paragraph on interactions	Authors
	Ligands (author curated)	A list of PubChem ligands with experiment type, nature of interactions, and Pubmed reference	Authors
	Interactions (author curated)	A list of interactors with experiment type, nature of interaction, and Pubmed reference	Authors
	Interactions (automatically populated)	A list of interactors with experiment type, nature of interaction, and Pubmed reference	BioGRID with input from author
	Transcriptional regulators (automatically populated)	A list of transcriptional regulators	PAZAR
Genetics	Genetics	A 250-word paragraph on genetics	Authors
	MeSH disease annotations (automatically populated)	Statistically significant associations with MeSH disease terms presented in cloud and table views, with Fisher's exact p-values	TFe, Medical Subject Headings
	MGI mammalian phenotype terms (automatically populated)	A list of associated MGI mammalian phenotype terms	Mouse Genome Database
Expression	Overview	A 200-word paragraph on genomic targets	Authors
	Expression (automatically populated)	Heat map of organism-wide expression data	UCSC Genome Browser
Ontologies	Gene Ontology (automatically populated)	Key associated GO terms presented as both a tree and a data table	Entrez Gene
	MeSH cloud (automatically populated)	Statistically significant associations with MeSH disease terms presented in cloud and table views, with Fisher's exact p-values	TFe, Medical Subject Headings
Papers	Papers	A list of relevant Pubmed papers	Authors
