# Supplementary material for: The Transcription Factor Encyclopedia
Source: Genome Biol. 2012 Mar 29;13(3):R24. doi: 10.1186/gb-2012-13-3-r24 (PMC3439975; doi:10.1186/gb-2012-13-3-r24)
Supplement: Additional file 5 — Binding models produced in the TFe project. Images of the binding models produced in TFe that are sufficiently characterized to be used in a study. [file gb-2012-13-3-r24-S5.PDF]

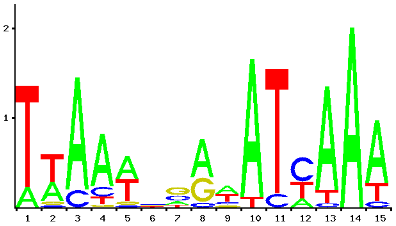

ARID3a (human)

AT rich interactive domain 3A (BRIGHT-like)

Helix-Turn-Helix Group, Arid Domain Family. PAZAR TF0000816. TFe 523.

PFM	1	2	3	4	5	6	7	8	9	10	11	12	13	14	15
A	5	11	26	22	14	6	5	17	12	28	0	5	26	30	22
C	0	1	4	4	1	9	9	2	4	0	3	15	1	0	2
G	0	2	0	1	2	8	13	11	3	0	0	0	0	0	0
T	25	16	0	3	13	7	3	0	11	2	27	10	3	0	6

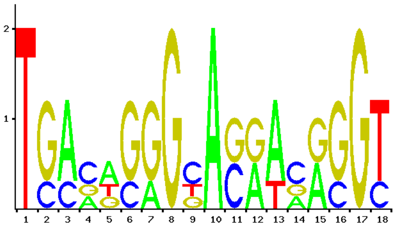

Arnt (mouse)

aryl hydrocarbon receptor nuclear translocator

Zipper-Type Group, Helix-Loop-Helix Family. PAZAR TF0000888. TFe 580.

PFM	1	2	3	4	5	6	7	8	9	10	11	12	13	14	15	16	17	18
A	0	0	3	1	2	0	1	0	0	4	0	2	3	1	2	0	0	0
C	0	1	1	2	0	1	0	0	2	0	2	0	0	2	0	1	0	1
G	0	3	0	1	1	3	3	4	1	0	2	2	0	1	2	3	4	0
T	4	0	0	0	1	0	0	0	1	0	0	0	1	0	0	0	0	3

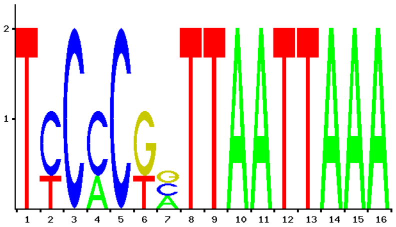

Arx (mouse)

aristaless related homeobox

Helix-Turn-Helix Group, Homeodomain Family. PAZAR TF0000800. TFe 583.

PFM	1	2	3	4	5	6	7	8	9	10	11	12	13	14	15	16
A	0	0	0	1	0	0	1	0	0	3	3	0	0	3	3	3
C	0	2	3	2	3	0	1	0	0	0	0	0	0	0	0	0
G	0	0	0	0	0	2	1	0	0	0	0	0	0	0	0	0
T	3	1	0	0	0	1	0	3	3	0	0	3	3	0	0	0

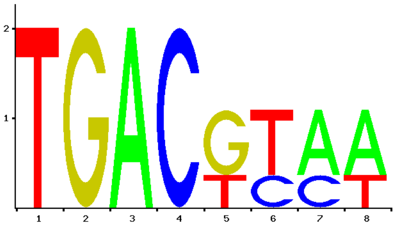

Atf2 (mouse)

activating transcription factor 2

Zipper-Type Group, Leucine Zipper Family. PAZAR TF0000883. TFe 584.

PFM	1	2	3	4	5	6	7	8
A	0	0	3	0	0	0	2	2
C	0	0	0	3	0	1	1	0
G	0	3	0	0	2	0	0	0
T	3	0	0	0	1	2	0	1

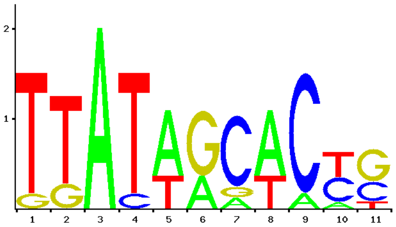

CDX2 (human)

caudal type homeobox 2

Helix-Turn-Helix Group, Homeodomain Family. PAZAR TF0000770. TFe 526.

PFM	1	2	3	4	5	6	7	8	9	10	11
A	0	0	9	0	6	3	1	6	1	1	0
C	0	0	0	1	0	0	7	0	8	4	3
G	1	2	0	0	0	6	1	0	0	0	5
T	8	7	0	8	3	0	0	3	0	4	1

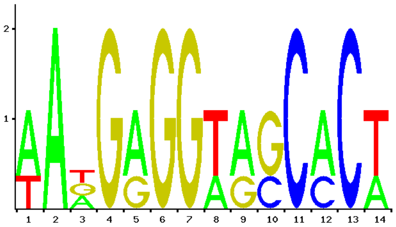

Arntl (mouse)

aryl hydrocarbon receptor nuclear translocator-like

Zipper-Type Group, Helix-Loop-Helix Family. PAZAR TF0000885. TFe 582.

PFM	1	2	3	4	5	6	7	8	9	10	11	12	13	14
A	2	3	1	0	2	0	0	1	2	0	0	2	0	1
C	0	0	0	0	0	0	0	0	0	1	3	1	3	0
G	0	0	1	3	1	3	3	0	1	2	0	0	0	0
T	1	0	1	0	0	0	0	2	0	0	0	0	0	2

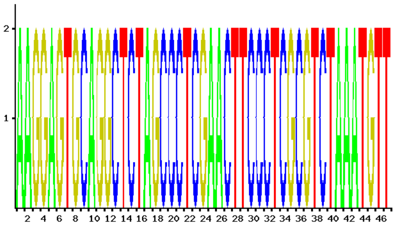

Ctcf (mouse)

CCCTC-binding factor

Zinc-coordinating Group, BetaBetaAlpha-zinc Finger Family. PAZAR TF0000997. TFe 613.

The position frequency matrix (PFM) for this logo is too large to be displayed in this area. Please visit the article on the TFe website at http://www.cisreg.ca/cgi-bin/tfe/articles.pl?tfid=613 to view the matrix and other information regarding this transcription factor.

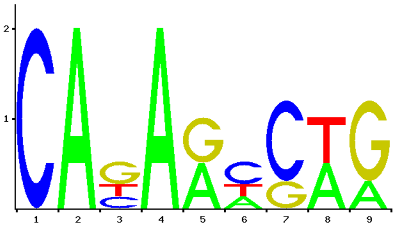

Foxa1 (mouse)

forkhead box A1

Winged Helix-Turn-Helix, Forkhead Domain Family. PAZAR TF0000716. TFe 172.

PFM	1	2	3	4	5	6	7	8	9
A	0	4	0	4	2	1	0	2	1
C	4	0	1	0	0	2	3	0	0
G	0	0	2	0	2	0	1	0	3
T	0	0	1	0	0	1	0	2	0

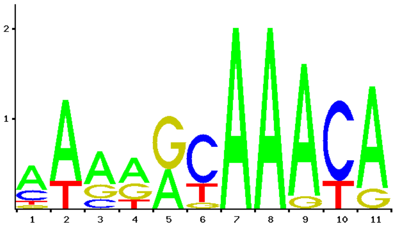

Foxa1 (mouse)

forkhead box A1

Winged Helix-Turn-Helix, Forkhead Domain Family. PAZAR TF0000718. TFe 172.

PFM	1	2	3	4	5	6	7	8	9	10	11
A	7	9	7	6	5	0	12	12	11	0	10
C	3	0	2	0	0	8	0	0	0	9	0
G	1	0	3	4	7	1	0	0	1	0	2
T	1	3	0	2	0	3	0	0	0	3	0

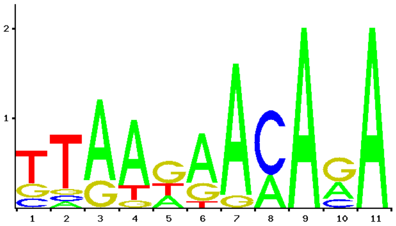

Foxa2 (rat)

forkhead box A2

Winged Helix-Turn-Helix, Forkhead Domain Family. PAZAR TF0000681. TFe 168.

PFM	1	2	3	4	5	6	7	8	9	10	11
A	0	1	9	9	3	8	11	4	12	4	12
C	2	1	0	0	0	0	0	8	0	2	0
G	3	1	3	1	6	3	1	0	0	6	0
T	7	9	0	2	3	1	0	0	0	0	0

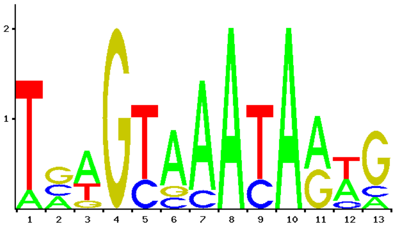

Foxa2 (mouse)

forkhead box A2

Winged Helix-Turn-Helix, Forkhead Domain Family. PAZAR TF0000717. TFe 167.

PFM	1	2	3	4	5	6	7	8	9	10	11	12	13
A	1	2	4	0	0	5	6	7	0	7	4	3	1
C	0	2	0	0	2	1	1	0	2	0	0	1	1
G	0	3	1	7	0	1	0	0	0	0	3	0	5
T	6	0	2	0	5	0	0	0	5	0	0	3	0

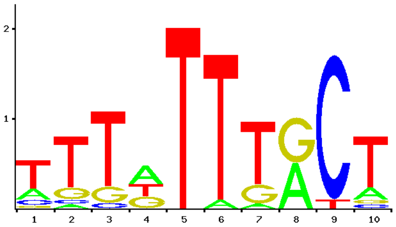

Foxa2 (mouse)

forkhead box A2

Winged Helix-Turn-Helix, Forkhead Domain Family. PAZAR TF0000720. TFe 167.

PFM	1	2	3	4	5	6	7	8	9	10
A	4	1	0	8	0	1	1	9	0	3
C	2	1	1	0	0	0	0	0	17	1
G	1	3	3	5	0	0	4	9	0	1
T	11	13	14	5	18	17	13	0	1	13

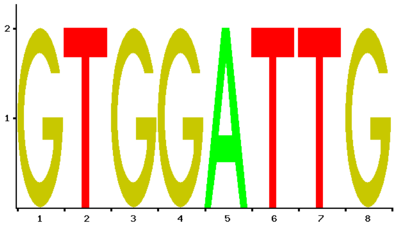

Foxh1 (mouse)

forkhead box H1

Winged Helix-Turn-Helix, Forkhead Domain Family. PAZAR TF0000679. TFe 171.

PFM	1	2	3	4	5	6	7	8
A	0	0	0	0	4	0	0	0
C	0	0	0	0	0	0	0	0
G	4	0	4	4	0	0	0	4
T	0	4	0	0	0	4	4	0

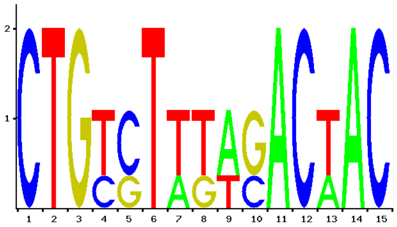

FOXL2 (human)

forkhead box L2

Winged Helix-Turn-Helix, Forkhead Domain Family. PAZAR TF0000786. TFe 436.

PFM	1	2	3	4	5	6	7	8	9	10	11	12	13	14	15
A	0	0	0	0	0	0	1	0	2	0	3	0	1	3	0
C	3	0	0	1	2	0	0	0	0	1	0	3	0	0	3
G	0	0	3	0	1	0	0	1	0	2	0	0	0	0	0
T	0	3	0	2	0	3	2	2	1	0	0	0	2	0	0

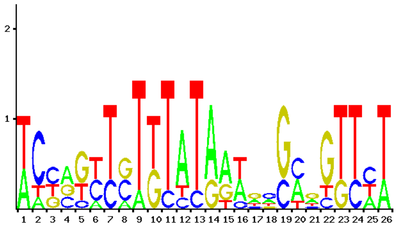

FOXM1 (human)

forkhead box M1

Winged Helix-Turn-Helix, Forkhead Domain Family. PAZAR TF0000881. TFe 437.

PFM	1	2	3	4	5	6	7	8	9	10	11	12	13	14	15	16	17	18	19	20	21	22	23	24	25	26
A	3	1	0	3	0	1	0	1	1	0	0	5	0	5	4	3	2	2	0	3	2	0	0	0	2	2
C	0	5	3	2	1	3	2	3	0	0	1	1	1	0	0	1	1	3	2	3	1	1	0	2	3	0
G	0	0	2	2	4	0	0	3	0	3	0	0	0	2	2	0	3	1	5	0	3	5	2	0	0	0
T	4	1	2	0	2	3	5	0	6	4	6	1	6	0	1	3	1	1	0	1	1	1	5	5	2	5

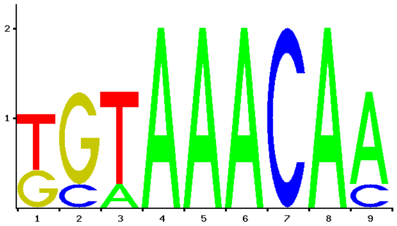

FOXO3 (human)

forkhead box O3

Winged Helix-Turn-Helix, Forkhead Domain Family. PAZAR TF0000811. TFe 443.

PFM	1	2	3	4	5	6	7	8	9
A	0	0	1	5	5	5	0	5	4
C	0	1	0	0	0	0	5	0	1
G	2	4	0	0	0	0	0	0	0
T	3	0	4	0	0	0	0	0	0

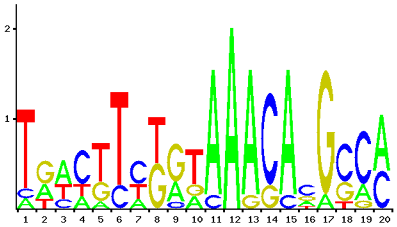

FOXO1 (human)

forkhead box O1

Winged Helix-Turn-Helix, Forkhead Domain Family. PAZAR TF0000812. TFe 442.

PFM	1	2	3	4	5	6	7	8	9	10	11	12	13	14	15	16	17	18	19	20
A	1	3	5	2	1	0	2	0	3	2	9	10	9	0	9	2	1	0	2	6
C	1	0	2	6	0	2	5	0	1	0	1	0	0	8	1	5	0	6	7	4
G	0	5	0	0	3	0	0	5	6	2	0	0	1	2	0	2	9	3	1	0
T	8	2	3	2	6	8	3	5	0	6	0	0	0	0	0	1	0	1	0	0

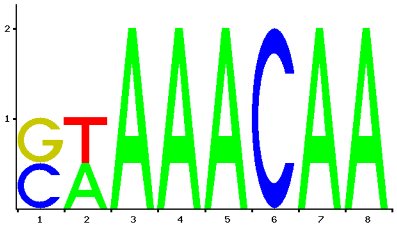

FOXO4 (human)

forkhead box O4

Winged Helix-Turn-Helix, Forkhead Domain Family. PAZAR TF0000815. TFe 444.

PFM	1	2	3	4	5	6	7	8
A	0	1	2	2	2	0	2	2
C	1	0	0	0	0	2	0	0
G	1	0	0	0	0	0	0	0
T	0	1	0	0	0	0	0	0

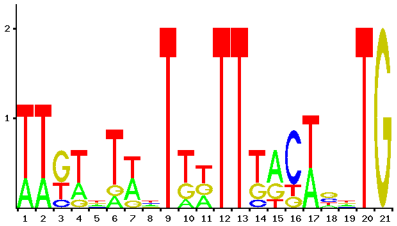

FOXO4 (human)

forkhead box O4

Winged Helix-Turn-Helix, Forkhead Domain Family. PAZAR TF0000771. TFe 444.

PFM	1	2	3	4	5	6	7	8	9	10	11	12	13	14	15	16	17	18	19	20	21
A	2	2	0	2	2	1	3	2	0	1	2	0	0	0	4	0	3	1	2	0	0
C	0	0	1	0	1	0	0	2	0	0	0	0	0	1	0	5	0	2	1	0	0
G	0	0	4	1	2	1	1	1	0	2	2	0	0	2	2	1	0	3	2	0	7
T	5	5	2	4	2	5	3	2	7	4	3	7	7	4	1	1	4	1	2	7	0

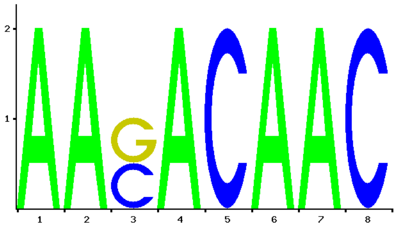

Foxp1 (mouse)

forkhead box P1

Winged Helix-Turn-Helix, Forkhead Domain Family. PAZAR TF0000805. TFe 480.

PFM	1	2	3	4	5	6	7	8
A	2	2	0	2	0	2	2	0
C	0	0	1	0	2	0	0	2
G	0	0	1	0	0	0	0	0
T	0	0	0	0	0	0	0	0

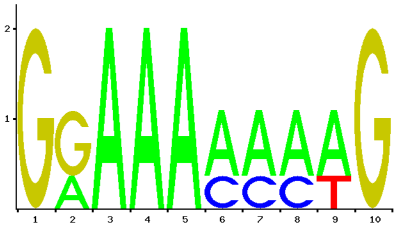

Foxp1 (mouse)

forkhead box P1

Winged Helix-Turn-Helix, Forkhead Domain Family. PAZAR TF0000802. TFe 480.

PFM	1	2	3	4	5	6	7	8	9	10
A	0	1	3	3	3	2	2	2	2	0
C	0	0	0	0	0	1	1	1	0	0
G	3	2	0	0	0	0	0	0	0	3
T	0	0	0	0	0	0	0	0	1	0

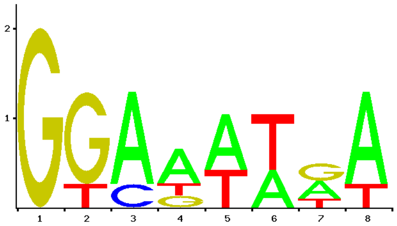

HHEX (human)

hematopoietically expressed homeobox

Not specified, Not specified. PAZAR TF0000803. TFe 558.

PFM	1	2	3	4	5	6	7	8
A	0	0	4	3	3	2	2	4
C	0	0	1	0	0	0	0	0
G	5	4	0	1	0	0	2	0
T	0	1	0	1	2	3	1	1

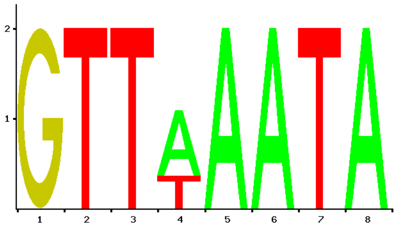

HNF1B (human)

HNF1 homeobox B

Helix-Turn-Helix Group, Homeodomain Family. PAZAR TF0000782. TFe 544.

PFM	1	2	3	4	5	6	7	8
A	0	0	0	2	3	3	0	3
C	0	0	0	0	0	0	0	0
G	3	0	0	0	0	0	0	0
T	0	3	3	1	0	0	3	0

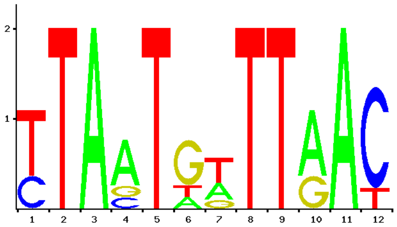

HNF1B (human)

HNF1 homeobox B

Helix-Turn-Helix Group, Homeodomain Family. PAZAR TF0000780. TFe 544.

PFM	1	2	3	4	5	6	7	8	9	10	11	12
A	0	0	6	4	0	1	2	0	0	4	6	0
C	2	0	0	1	0	0	0	0	0	0	0	5
G	0	0	0	1	0	4	1	0	0	2	0	0
T	4	6	0	0	6	1	3	6	6	0	0	1

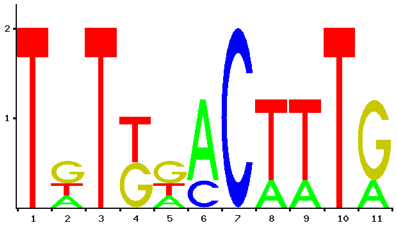

HNF4G (human)

hepatocyte nuclear factor 4, gamma

Zinc-coordinating Group, Hormone-nuclear Receptor Family. PAZAR TF0000900. TFe 310.

PFM	1	2	3	4	5	6	7	8	9	10	11
A	0	1	0	0	1	3	0	1	1	0	1
C	0	0	0	0	0	1	4	0	0	0	0
G	0	2	0	2	2	0	0	0	0	0	3
T	4	1	4	2	1	0	0	3	3	4	0

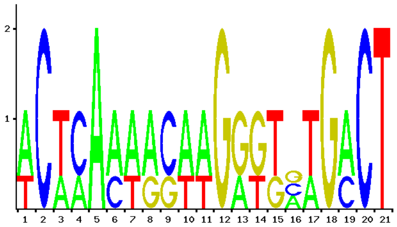

HNF4G (human)

hepatocyte nuclear factor 4, gamma

Zinc-coordinating Group, Hormone-nuclear Receptor Family. PAZAR TF0000903. TFe 310.

PFM	1	2	3	4	5	6	7	8	9	10	11	12	13	14	15	16	17	18	19	20	21
A	2	0	1	1	3	2	2	2	0	2	2	0	1	0	0	1	1	0	2	0	0
C	0	3	0	2	0	1	0	0	2	0	0	0	0	0	0	1	0	0	1	3	0
G	0	0	0	0	0	0	0	1	1	0	0	3	2	2	1	1	0	3	0	0	0
T	1	0	2	0	0	0	1	0	0	1	1	0	0	1	2	0	2	0	0	0	3

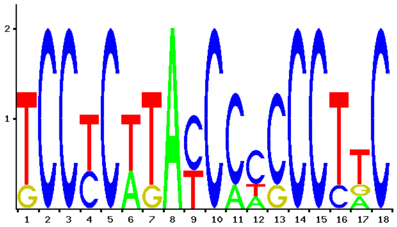

Hnrnpk (mouse)

heterogeneous nuclear ribonucleoprotein K

Not specified, Not specified. PAZAR TF0000778. TFe 552.

PFM	1	2	3	4	5	6	7	8	9	10	11	12	13	14	15	16	17	18
A	0	0	0	0	0	2	0	5	0	0	1	1	0	0	0	0	1	0
C	0	5	5	2	5	0	0	0	3	5	4	3	4	5	5	1	0	5
G	1	0	0	0	0	0	1	0	0	0	0	0	1	0	0	0	1	0
T	4	0	0	3	0	3	4	0	2	0	0	1	0	0	0	4	3	0

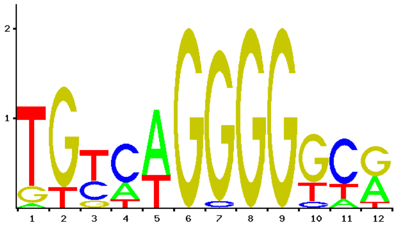

INSM1 (human)

insulinoma-associated 1

Not specified, Not specified. PAZAR TF0000773. TFe 548.

PFM	1	2	3	4	5	6	7	8	9	10	11	12
A	1	0	0	6	16	0	0	0	0	0	3	10
C	0	0	8	15	0	0	1	0	0	2	16	0
G	4	20	3	0	0	24	23	24	24	16	0	12
T	19	4	13	3	8	0	0	0	0	6	5	2

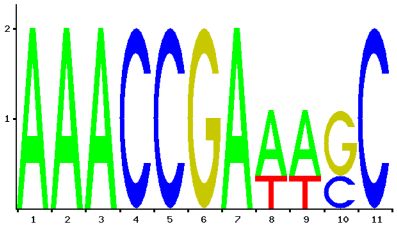

Irf1 (mouse)

interferon regulatory factor 1

Winged Helix-Turn-Helix, Interferon Regulatory Factor. PAZAR TF0000918. TFe 703.

PFM	1	2	3	4	5	6	7	8	9	10	11
A	3	3	3	0	0	0	3	2	2	0	0
C	0	0	0	3	3	0	0	0	0	1	3
G	0	0	0	0	0	3	0	0	0	2	0
T	0	0	0	0	0	0	0	1	1	0	0

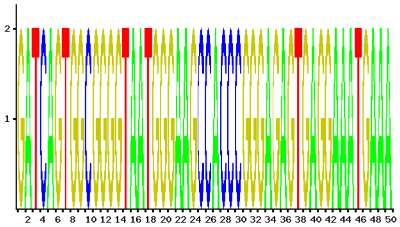

Irf1 (mouse)

interferon regulatory factor 1

Winged Helix-Turn-Helix, Interferon Regulatory Factor. PAZAR TF0001101. TFe 703.

The position frequency matrix (PFM) for this logo is too large to be displayed in this area. Please visit the article on the TFe website at http://www.cisreg.ca/cgi-bin/tfe/articles.pl?tfid=703 to view the matrix and other information regarding this transcription factor.

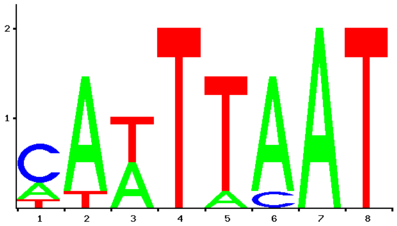

LHX3 (human)

LIM homeobox 3

Helix-Turn-Helix Group, Homeodomain Family. PAZAR TF0000801. TFe 515.

PFM	1	2	3	4	5	6	7	8
A	2	7	4	0	1	7	8	0
C	5	0	0	0	0	1	0	0
G	0	0	0	0	0	0	0	0
T	1	1	4	8	7	0	0	7

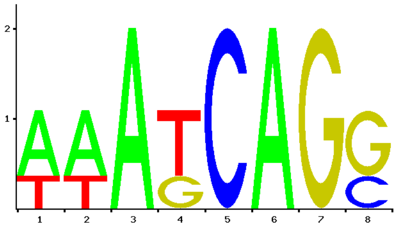

LHX3 (human)

LIM homeobox 3

Helix-Turn-Helix Group, Homeodomain Family. PAZAR TF0000836. TFe 515.

PFM	1	2	3	4	5	6	7	8
A	2	2	3	0	0	3	0	0
C	0	0	0	0	3	0	0	1
G	0	0	0	1	0	0	3	2
T	1	1	0	2	0	0	0	0

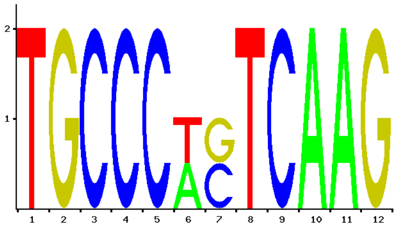

Nkx2-1 (mouse)

NK2 homeobox 1

Helix-Turn-Helix Group, Homeodomain Family. PAZAR TF0000834. TFe 888.

PFM	1	2	3	4	5	6	7	8	9	10	11	12
A	0	0	0	0	0	1	0	0	0	2	2	0
C	0	0	2	2	2	0	1	0	2	0	0	0
G	0	2	0	0	0	0	1	0	0	0	0	2
T	2	0	0	0	0	1	0	2	0	0	0	0

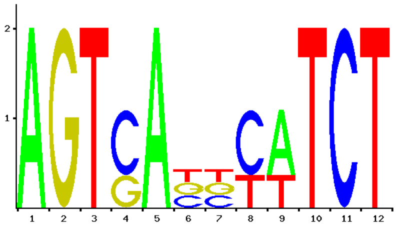

Nr2e1 (mouse)

nuclear receptor subfamily 2, group E, member 1

Zinc-coordinating Group, Hormone-nuclear Receptor Family. PAZAR TF0000733. TFe 181.

PFM	1	2	3	4	5	6	7	8	9	10	11	12
A	3	0	0	0	3	0	0	0	2	0	0	0
C	0	0	0	2	0	1	1	2	0	0	3	0
G	0	3	0	1	0	1	1	0	0	0	0	0
T	0	0	3	0	0	1	1	1	1	3	0	3

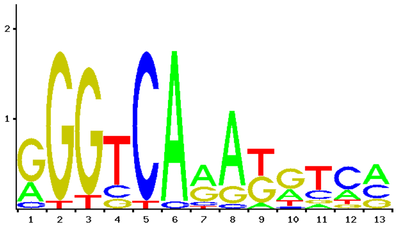

NR2F2 (human)

nuclear receptor subfamily 2, group F, member 2

Zinc-coordinating Group, Hormone-nuclear Receptor Family. PAZAR TF0000781. TFe 323.

PFM	1	2	3	4	5	6	7	8	9	10	11	12	13
A	6	0	0	0	0	21	11	17	2	6	2	5	10
C	2	0	0	4	21	1	2	1	0	1	5	13	7
G	14	21	20	3	0	0	8	4	10	12	2	2	5
T	0	1	2	15	1	0	1	0	10	3	13	2	0

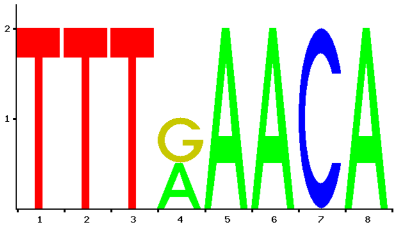

PAX6 (human)

paired box 6

Helix-Turn-Helix Group, Homeodomain Family. PAZAR TF0000994. TFe 506.

PFM	1	2	3	4	5	6	7	8
A	0	0	0	1	2	2	0	2
C	0	0	0	0	0	0	2	0
G	0	0	0	1	0	0	0	0
T	2	2	2	0	0	0	0	0

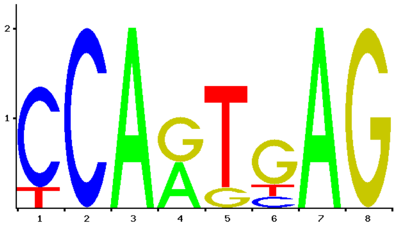

PAX6 (human)

paired box 6

Helix-Turn-Helix Group, Homeodomain Family. PAZAR TF0000993. TFe 506.

PFM	1	2	3	4	5	6	7	8
A	0	0	6	3	0	0	6	0
C	5	6	0	0	0	1	0	0
G	0	0	0	3	1	4	0	6
T	1	0	0	0	5	1	0	0

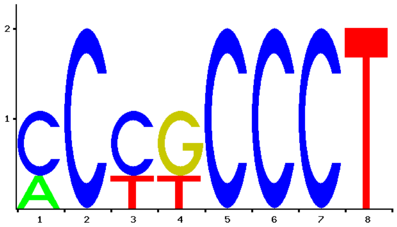

Pax8 (mouse)

paired box gene 8

Helix-Turn-Helix Group, Homeodomain Family. PAZAR TF0000832. TFe 793.

PFM	1	2	3	4	5	6	7	8
A	1	0	0	0	0	0	0	0
C	2	3	2	0	3	3	3	0
G	0	0	0	2	0	0	0	0
T	0	0	1	1	0	0	0	3

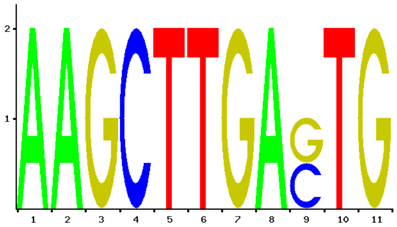

Pax8 (mouse)

paired box gene 8

Helix-Turn-Helix Group, Homeodomain Family. PAZAR TF0000831. TFe 793.

PFM	1	2	3	4	5	6	7	8	9	10	11
A	2	2	0	0	0	0	0	2	0	0	0
C	0	0	0	2	0	0	0	0	1	0	0
G	0	0	2	0	0	0	2	0	1	0	2
T	0	0	0	0	2	2	0	0	0	2	0

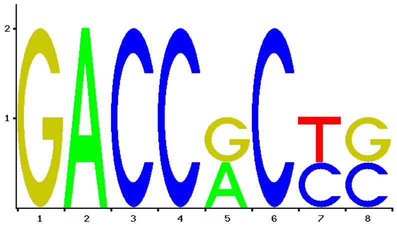

Pdx1 (mouse)

pancreatic and duodenal homeobox 1

Helix-Turn-Helix Group, Homeodomain Family. PAZAR TF0000920. TFe 797.

PFM	1	2	3	4	5	6	7	8
A	0	2	0	0	1	0	0	0
C	0	0	2	2	0	2	1	1
G	2	0	0	0	1	0	0	1
T	0	0	0	0	0	0	1	0

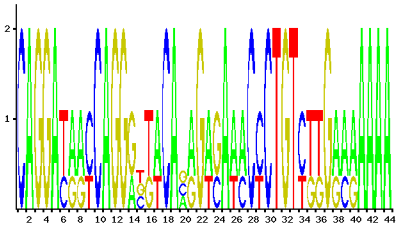

Prdm1 (mouse)

PR domain containing 1, with ZNF domain

Zinc-coordinating Group, BetaBetaAlpha-zinc Finger Family. PAZAR TF0001103. TFe 596.

The position frequency matrix (PFM) for this logo is too large to be displayed in this area. Please visit the article on the TFe website at http://www.cisreg.ca/cgi-bin/tfe/articles.pl?tfid=596 to view the matrix and other information regarding this transcription factor.

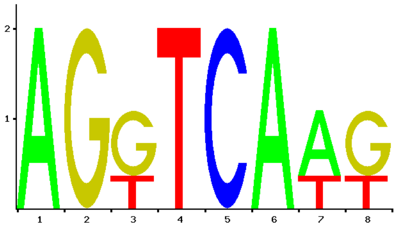

Ppara (mouse)

peroxisome proliferator activated receptor alpha

Zinc-coordinating Group, Hormone-nuclear Receptor Family. PAZAR TF0000895. TFe 384.

PFM	1	2	3	4	5	6	7	8
A	3	0	0	0	0	3	2	0
C	0	0	0	0	3	0	0	0
G	0	3	2	0	0	0	0	2
T	0	0	1	3	0	0	1	1

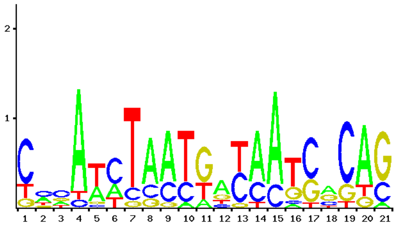

Six3 (mouse)

sine oculis-related homeobox 3 homolog (Drosophila)

Helix-Turn-Helix Group, Homeodomain Family. PAZAR TF0000841. TFe 489.

PFM	1	2	3	4	5	6	7	8	9	10	11	12	13	14	15	16	17	18	19	20	21
A	0	5	3	13	5	4	0	10	10	1	2	8	0	10	12	1	0	6	0	11	1
C	10	6	7	1	1	8	2	3	4	4	0	2	7	4	3	1	9	3	11	0	4
G	2	2	3	0	1	0	1	2	1	0	9	3	1	0	0	4	5	5	3	2	10
T	3	2	2	1	8	3	12	0	0	10	4	2	7	1	0	9	1	1	1	2	0

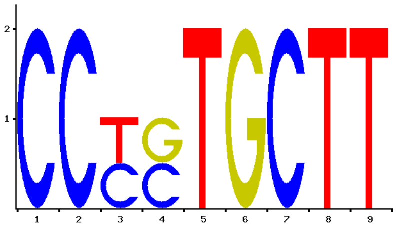

Six6 (mouse)

sine oculis-related homeobox 6 homolog (Drosophila)

Helix-Turn-Helix Group, Homeodomain Family. PAZAR TF0000884. TFe 491.

PFM	1	2	3	4	5	6	7	8	9
A	0	0	0	0	0	0	0	0	0
C	2	2	1	1	0	0	2	0	0
G	0	0	0	1	0	2	0	0	0
T	0	0	1	0	2	0	0	2	2

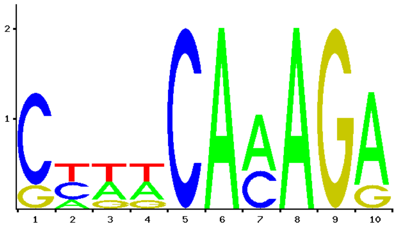

Sox10 (mouse)

SRY-box containing gene 10

Other Alpha-Helix Group, High Mobility Group (Box) Family. PAZAR TF0000905. TFe 497.

PFM	1	2	3	4	5	6	7	8	9	10
A	0	1	2	2	0	5	3	5	0	4
C	4	2	0	0	5	0	2	0	0	0
G	1	0	1	1	0	0	0	0	5	1
T	0	2	2	2	0	0	0	0	0	0

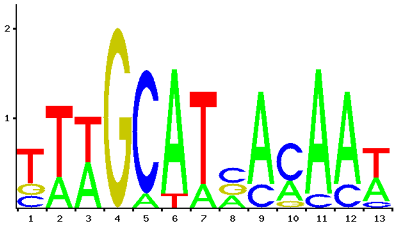

Sox2 (mouse)

SRY-box containing gene 2

Other Alpha-Helix Group, High Mobility Group (Box) Family. PAZAR TF0000779. TFe 531.

PFM	1	2	3	4	5	6	7	8	9	10	11	12	13
A	0	3	5	0	1	9	2	3	8	3	9	8	4
C	2	0	0	0	9	0	0	4	2	6	1	2	1
G	2	0	0	10	0	0	0	3	0	1	0	0	0
T	6	7	5	0	0	1	8	0	0	0	0	0	5

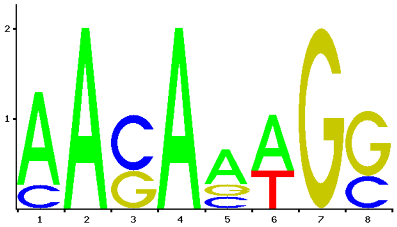

Sox8 (mouse)

SRY-box containing gene 8

Other Alpha-Helix Group, High Mobility Group (Box) Family. PAZAR TF0000892. TFe 493.

PFM	1	2	3	4	5	6	7	8
A	4	5	0	5	3	3	0	0
C	1	0	3	0	1	0	0	1
G	0	0	2	0	1	0	5	2
T	0	0	0	0	0	2	0	0

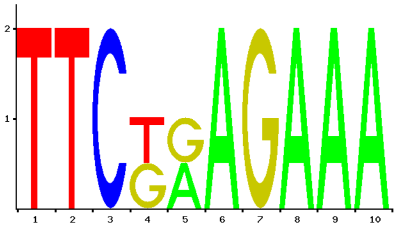

Stat5b (mouse)

signal transducer and activator of transcription 5B

Other, Stat Protein Family. PAZAR TF0000687. TFe 852.

PFM	1	2	3	4	5	6	7	8	9	10
A	0	0	0	0	1	2	0	2	2	2
C	0	0	2	0	0	0	0	0	0	0
G	0	0	0	1	1	0	2	0	0	0
T	2	2	0	1	0	0	0	0	0	0

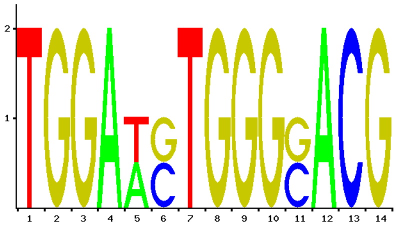

FOXO3 (human)

forkhead box O3

Winged Helix-Turn-Helix, Forkhead Domain Family. PAZAR TF0000810. TFe 443.

PFM	1	2	3	4	5	6	7	8	9	10	11	12	13	14
A	0	0	0	2	1	0	0	0	0	0	0	2	0	0
C	0	0	0	0	0	1	0	0	0	0	1	0	2	0
G	0	2	2	0	0	1	0	2	2	2	1	0	0	2
T	2	0	0	0	1	0	2	0	0	0	0	0	0	0

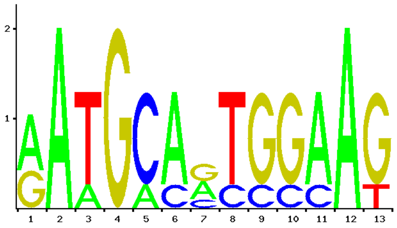

TWIST1 (human)

twist homolog 1 (Drosophila)

Zipper-Type Group, Helix-Loop-Helix Family. PAZAR TF0000909. TFe 1030.

PFM	1	2	3	4	5	6	7	8	9	10	11	12	13
A	3	5	1	0	1	4	2	0	0	0	4	5	0
C	0	0	0	0	4	1	1	1	1	1	1	0	0
G	2	0	0	5	0	0	2	0	4	4	0	0	4
T	0	0	4	0	0	0	0	4	0	0	0	0	1

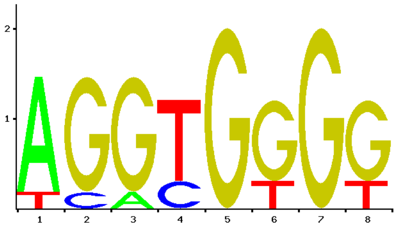

TWIST1 (human)

twist homolog 1 (Drosophila)

Zipper-Type Group, Helix-Loop-Helix Family. PAZAR TF0000886. TFe 1030.

PFM	1	2	3	4	5	6	7	8
A	7	0	1	0	0	0	0	0
C	0	1	0	2	0	0	0	0
G	0	7	7	0	8	6	8	6
T	1	0	0	6	0	2	0	2

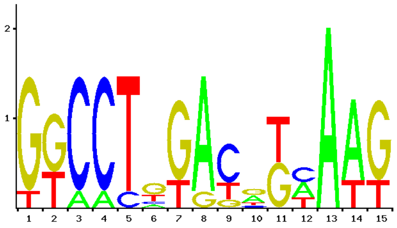

ZBTB16 (human)

zinc finger and BTB domain containing 16

Not specified, Not specified. PAZAR TF0000764. TFe 413.

PFM	1	2	3	4	5	6	7	8	9	10	11	12	13	14	15
A	0	0	1	1	0	1	0	7	0	3	0	3	8	6	0
C	0	0	7	7	1	1	0	0	5	1	0	3	0	0	0
G	7	5	0	0	0	4	6	1	1	3	4	0	0	0	6
T	1	3	0	0	7	2	2	0	2	1	4	2	0	2	2

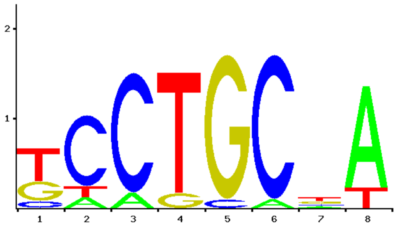

Zbtb33 (mouse)

zinc finger and BTB domain containing 33

Zinc-coordinating Group, BetaBetaAlpha-zinc Finger Family. PAZAR TF0000910. TFe 944.

PFM	1	2	3	4	5	6	7	8
A	0	2	2	0	0	1	3	15
C	2	14	16	0	1	17	3	0
G	6	0	0	2	17	0	5	0
T	10	2	0	16	0	0	7	3

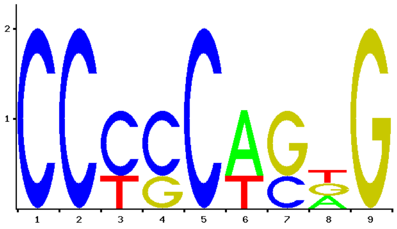

Zic1 (mouse)

zinc finger protein of the cerebellum 1

Zinc-coordinating Group, BetaBetaAlpha-zinc Finger Family. PAZAR TF0000898. TFe 570.

PFM	1	2	3	4	5	6	7	8	9
A	0	0	0	0	0	2	0	1	0
C	3	3	2	2	3	0	1	0	0
G	0	0	0	1	0	0	2	1	3
T	0	0	1	0	0	1	0	1	0

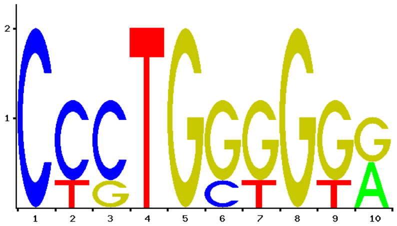

Zic2 (mouse)

zinc finger protein of the cerebellum 2

Zinc-coordinating Group, BetaBetaAlpha-zinc Finger Family. PAZAR TF0000799. TFe 571.

PFM	1	2	3	4	5	6	7	8	9	10
A	0	0	0	0	0	0	0	0	0	2
C	4	3	3	0	0	1	0	0	0	0
G	0	0	1	0	4	3	3	4	3	2
T	0	1	0	4	0	0	1	0	1	0
